# Standardizing protocols for determining the cause of mortality in wildlife studies

**DOI:** 10.1002/ece3.9034

**Published:** 2022-06-23

**Authors:** Bogdan Cristescu, L. Mark Elbroch, Tavis D. Forrester, Maximilian L. Allen, Derek B. Spitz, Christopher C. Wilmers, Heiko U. Wittmer

**Affiliations:** ^1^ Environmental Studies Department University of California Santa Cruz California USA; ^2^ Panthera New York New York USA; ^3^ Oregon Department of Fish and Wildlife Wildlife Research La Grande Oregon USA; ^4^ Illinois Natural History Survey University of Illinois Champaign Illinois USA; ^5^ School of Biological Sciences Victoria University of Wellington Wellington New Zealand

**Keywords:** cause of death, kill site, mule deer, predation, survival analysis, temperate ecosystem, ungulate neonate

## Abstract

Mortality site investigations of telemetered wildlife are important for cause‐specific survival analyses and understanding underlying causes of observed population dynamics. Yet, eroding ecoliteracy and a lack of quality control in data collection can lead researchers to make incorrect conclusions, which may negatively impact management decisions for wildlife populations. We reviewed a random sample of 50 peer‐reviewed studies published between 2000 and 2019 on survival and cause‐specific mortality of ungulates monitored with telemetry devices. This concise review revealed extensive variation in reporting of field procedures, with many studies omitting critical information for the cause of mortality inference. Field protocols used to investigate mortality sites and ascertain the cause of mortality are often minimally described and frequently fail to address how investigators dealt with uncertainty. We outline a step‐by‐step procedure for mortality site investigations of telemetered ungulates, including evidence that should be documented in the field. Specifically, we highlight data that can be useful to differentiate predation from scavenging and more conclusively identify the predator species that killed the ungulate. We also outline how uncertainty in identifying the cause of mortality could be acknowledged and reported. We demonstrate the importance of rigorous protocols and prompt site investigations using data from our 5‐year study on survival and cause‐specific mortality of telemetered mule deer (*Odocoileus hemionus*) in northern California. Over the course of our study, we visited mortality sites of neonates (*n* = 91) and adults (*n* = 23) to ascertain the cause of mortality. Rapid site visitations significantly improved the successful identification of the cause of mortality and confidence levels for neonates. We discuss the need for rigorous and standardized protocols that include measures of confidence for mortality site investigations. We invite reviewers and journal editors to encourage authors to provide supportive information associated with the identification of causes of mortality, including uncertainty.

## Graphical abstract

‐ Mortality site investigations provide critical data for population ecology, but the complexities of field investigations and confounding factors can affect replicability, and potentially lead to unreliable conclusions and management recommendations. ‐ We elaborate on common problems and on how sampling methods can be improved, summarize essential information to be recorded, and introduce a simple means to account for uncertainty in a decision tree approach. ‐ We illustrate our points with data from an intensive study on ungulate survival in California, in a system with multiple carnivores.

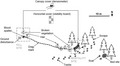

## 1 INTRODUCTION

Ecological research is inherently data driven and has been traditionally based on direct observations collected in the field. There is, however, cause for concern that eroding ecoliteracy surrounding the study of organisms and their linkages to the environment is impacting our ability to conduct accurate ecological research (Middendorf & Pohlad, 2014; Tewksbury et al., 2014). For example, quantifying vital rates such as age‐specific survival and estimating population size are fundamental to understanding the dynamics of animal populations (Caughley, 1994; Gaillard et al., 2000), but require accurate cause‐specific mortality parameters derived from field‐based evidence. While there have been many recent advances in methods used to accurately estimate vital rates and population size from various data sources (Silvy, 2012), little research focus is currently directed at the importance of correctly identifying the causes of observed mortalities of telemetered animals. Incorrectly categorizing causes of mortality could result in biased survival probabilities (Marescot et al., 2015) and carries management and conservation implications for both predators and prey (e.g., increased carnivore control and decreased ungulate hunting; Proffitt et al., 2020).

In North America, predation is sometimes identified as an underlying cause of decline for ungulate populations, often secondary to effects of weather and forage (Brodie et al., 2013; Forrester & Wittmer, 2013; Lukacs et al., 2018), or via apparent competition where the species impacted by predation is usually of conservation concern (e.g., Johnson et al., 2013; Wittmer et al., 2005). In addition, the debate as to whether increased predation following the recovery of many predator species in North America and Europe reduces opportunities for game hunting is ongoing (Forrester & Wittmer, 2013; Jonzén et al., 2013; Ripple et al., 2019). Given that the implementation of management actions such as habitat modification or increasingly controversial predator control are frequently based on analyses of survival and/or the identification of primary causes of mortality (Bergman et al., 2014; Hervieux et al., 2014), we are concerned by the often limited information associated with mortality investigations. We have three principal concerns with the cause of mortality data that management and conservation strategies for carnivores and prey are based upon: (1) the correct identification of predation events (i.e., differentiating predation from other causes of mortality); (2) the correct identification of the predatory species responsible for the mortality; and (3) the robustness of reporting the methodology used to derive 1 and 2.

Recent advances in telemetry technology are enabling researchers to remotely monitor many activities of wild animals and to more precisely determine the timing of an animal's death (Hebblewhite & Haydon, 2010; Wilmers et al., 2015). Current data loggers, however, do not transmit data on cause of death and, until cameras are routinely integrated into monitoring devices and are capable of storing large amounts of data (e.g., Thompson et al., 2012), researchers continue to depend on detailed site investigations to determine the cause of death. Correctly determining the probable cause of mortality, however, requires extensive experience (often years) that relies on excellent natural history knowledge and field skills to recognize and interpret animal signs (e.g., footprints and feces). As a result, even experienced wildlife professionals occasionally come to incorrect conclusions (Morin et al., 2016; Wysong et al., 2019). This suggests that a measure of confidence in mortality assessment can be informative. Yet because many academic institutions no longer prioritize field skills in their curricula, ecological research is increasingly driven by new cohorts of biologists who have received very little training in natural history and the relevant methods used in the field (Tewksbury et al., 2014). Furthermore, while wildlife agencies increasingly hire biologists with graduate degrees and direct field experience, not all graduate research projects involve intensive fieldwork and those that do may not have helped develop skills needed for investigating often complex predator–prey relationships in the field. The erosion of ecoliteracy (Middendorf & Pohlad, 2014) thus has the potential to inhibit the conducting of rigorous ecological research, and perhaps more importantly, the quality of data being analyzed to draw conclusions. In studies on prey survival, erroneous assessments of causes of mortality may result in overstating the effects of specific predator species and lead to the implementation of ineffective and controversial management strategies.

Even when field investigations of mortality sites are carried out based on strict protocols, researchers often include minimal information explaining how they determined the cause of death in the field and their level of confidence in their assigned cause of death. The lack of information regarding mortality assessments should raise concerns from reviewers during the publication process, but generally, it is not considered important enough for editors or reviewers to require authors to provide additional supporting information. As a consequence, there is both overconfidence and a lack of confidence in published results on causes of mortality, depending upon the wildlife professional and their familiarity with the difficulties associated with kill site investigations.

Perhaps the most difficult and contentious type of data is determining whether an ungulate was killed by a carnivore or died of another cause and was later scavenged. Further, the determination of which carnivore killed the ungulate is without doubt influenced by observer experience and internal biases, which may in turn be influenced by the expectations and biases of project leaders, funders or permitting agencies. Researchers can use environmental cues to help in the identification of the carnivore species responsible for mortalities of marked ungulates. For example, prey vulnerability and thus mortality site characteristics can differ in relation to habitat or vegetation characteristics among cursorial and ambush predators (Gorini et al., 2012), but this has limited application because many predators are generalists and utilize multiple habitats. Further, prey may be moved from kill to feeding sites in different habitats, as has been observed in several species (e.g., pumas; Allen et al., 2015). Genetic tools are also increasingly being employed in the identification of the carnivore species. Genetic analyses of the saliva collected at wound sites can determine the species responsible (Mumma et al., 2014), but the observer still must differentiate predation from scavenging.

Here, we have four major objectives: First, we summarize essential parameters and challenges associated with cause‐specific mortality research, determined via a concise review of a subset of literature reporting the cause of death for ungulates equipped with telemetry devices in North America or Europe. Second, we provide a step‐by‐step protocol for establishing the cause of mortality in the field for telemetered ungulates. Third, we highlight key aspects for critically evaluating predator‐specific field evidence in multi‐predator systems that may include felid, canid, and ursid predators, the most common carnivores preying on ungulates in North America and Europe. For the second and third objectives, we refer readers to some of the most relevant resources that expand on these issues and include a list of required tools, as well as data sheets and photographic references. Fourth, we draw on our experience with mortality site investigations for mule deer (*Odocoileus hemionus*) in northern California, USA, as a case study to illustrate how relevant information used to ascertain the cause of mortality should be collected and made available. We argue that being transparent with regard to monitoring regimes and the information used to identify the cause of mortality will reduce the probability of observational errors affecting study outcomes and conclusions. The considerations presented here are, therefore, pertinent to mortality investigations for all telemetered ungulates. Additional aspects of conducting kill site investigations based on GPS cluster analyses of marked carnivores, including their shortcomings, have been described elsewhere (Elbroch et al., 2018; Knopff et al., 2009; Merrill et al., 2010).

## 2 TERMINOLOGY

We use the following terms associated with mortality investigations. First, we use the term “mortality site” to broadly describe the location of either the entire carcass or significant parts thereof. For large prey species, this will usually be the site where the telemetry device is found, but we caution that some carnivores or scavengers move the telemetry unit away from the mortality site. The mortality site does not have to be equivalent to the “kill site,” which we define as the location an animal was subdued by a predator. Every effort should be undertaken to determine whether the mortality site and kill site are the same, since the kill site illustrates prey vulnerability and the feeding site is more determined by predator vulnerability (May et al., 2008). Our definition of mortality site includes feeding sites, which are often concealed habitats where the predator feels less exposed and thus comfortable to feed for extended periods of time. For some predator species, feeding sites will be closely associated with food caches containing all or part of the carcass in addition to other remains such as bones and hair (Elbroch & McFarland, 2019).

## 3 CURRENT APPROACHES FOR DETERMINING AND REPORTING THE CAUSE OF MORTALITY INFORMATION

Ungulate survival analyses dependent upon cause of mortality have received considerable attention in the scientific literature. A Google Scholar search (January 20, 2021) of “ungulate survival cause of mortality,” constrained to include all of these words anywhere in the article, yielded 29,875 records between 2000 and 2019. Based upon a linear regression analysis for time series across years, the number of records has consistently increased (β ± SE = 86.96 ± 3.24, *R* ^2^ = 0.98, *p* < .0001).

We selected 50 peer‐reviewed studies published in the above period (ungulate age class: *n*_neonate_ = 25, *n*_adult_ = 25), provided they occurred in North America or Europe and involved the use of telemetry to monitor study animals. We included one of our own studies (Marescot et al., 2015) for both neonate and adult age classes and used stratified random sampling to select the remaining 48 studies. Studies were selected randomly from Google Scholar outputs sorted by relevance, and ungulate species were assigned as strata to minimize over‐representation of widely studied species, with a maximum of five studies included per species within an ungulate age class. To avoid subjectivity, we did not inspect the Methods section of the studies as part of the selection process. We extracted a set of parameters from each study (Appendix S1: Tables S1 and S2) and summarized the main challenges herein. We wanted to explore the potential variability in methodological reporting in the selected article set, and we recognize that some studies that we did not review will fall outside the parameter values in Appendix S1. Our intent was to draw a sample from the literature to highlight methodological data challenges, rather than an exhaustive review of the cause of mortality studies.

We counted the number of words and in‐text citations dedicated to the mortality assessment protocol in the Methods section of articles, which served as qualitative measures of comprehensiveness in the description of field procedures. We focused on critical components of field analyses of the mortality site, carcass, and habitat, as well as on text that listed or detailed the assignment of cause of death from field investigations. The descriptions of transmitter monitoring regime, carcass pickup for lab necropsy, and sample collection and processing were not included in our word and citation counts but were listed in Appendix S1. Only 36% of studies described mortality site investigation procedures in ≥100 words, whereas 52% did so in <100 words and 12% (*n*_neonate_ = 1, *n*_adult_ = 5) did not specify any mortality assessment criteria. While some studies described procedures in detail, many (including one of our own, Marescot et al., 2015) provided only 1 to 2 sentences to explain mortality site investigations (Appendix S1: Tables S1 and S2). Sometimes authors only mentioned generically that the state and disposition of the carcass, tracks, and other signs were used to determine the cause of mortality, without further details or reference to protocols. Only 58% of studies provided in‐text citations for the mortality site investigation procedures, primarily for fawn studies (72%) and less so for adults (44%), whereas almost half of studies did not provide citations to justify the protocol (*n*_neonate_ = 7, *n*_adult_ = 14). Most studies without in‐text citations explained the protocol in <100 words (*n*_neonate_ = 6, *n*_adult_ = 9), with five studies not dedicating any text or citations to protocol description.

Twenty‐four percent of the ungulate survival studies that we reviewed (*n*_neonate_ = 3, *n*_adult_ = 9) did not specify the species composition of the predator community in their respective study areas. Of the 38 studies that specified the predator species present, 37 occurred in multi‐predator communities. In study systems that host complex predator and scavenger guilds, differentiating predation from scavenging and identifying the predator species that caused the mortality can be particularly challenging due to the possibility that several species might visit the carcass and/or are capable of making the kill.

Field evidence may, in some instances, be insufficient to make a reliable assessment of the cause of mortality. Lab necropsies were included on a case‐by‐case basis in less than half of the studies (42%), with most studies relying on field assessment alone (58%, *n*_neonate_ = 11, *n*_adult_ = 18). DNA was collected to identify predators in only 14% of the studies, all in studies involving neonates.

The time interval used for monitoring telemetered individuals, as well as how promptly researchers are able to visit the mortality site once the telemetry device was detected on mortality mode, most likely influence site investigation results. Overall, monitoring regimes were highly variable both within and among studies (Appendix S1: Tables S1, S2). A few studies (6%) did not report their telemetry monitoring regimes and one study assumed predation for all non‐hunting‐related mortalities, but other studies (46%, *n*_neonate_ = 18, *n*_adult_ = 5) had frequent relocation intervals (≥1/day) during specific periods. These focal periods included the first‐week post‐capture, the initial weeks of the neonates' lives, and/or when adults were tracked to locate and tag their offspring. For at least part of the time, some projects (34%, *n*_neonate_ = 6, *n*_adult_ = 11) monitored tagged individuals less frequently than weekly. The variation in monitoring regimes introduced additional uncertainty that made outcomes difficult to compare across study systems. Such comparisons were further complicated by differences in species composition and densities of complex multi‐predator and scavenger communities.

Most studies (94%) reported cause of mortality as “Unknown” when uncertain in the mortality assessment outcome, but for 6% of studies (*n*_neonate_ = 2, *n*_adult_ = 1) it was unclear whether mortalities were classified as “Unknown” when confronted with uncertainty. In addition, 16% of studies (*n*_neonate_ = 5, *n*_adult_ = 3) presented “Unknown” mortalities in a figure without specifying what percentage of “Unknown” mortalities were excluded from analyses. The definition of “Unknown” is contentious as well, given that the classification depends upon different confidence thresholds for different observers (i.e., some people may say "Unknown" only if 10% uncertain about the cause of death, and others 50%). One study acknowledged that identifying mortality causes was unreliable due to extensive time elapsed between mortality and site visitation but did not report what percentage each cause of death contributed to overall mortality.

## 4 A PROPOSED MORTALITY SITE INVESTIGATION PROTOCOL

Some agencies and research programs have manuals or field guides to interpret signs at mortality sites for determining the cause of death, typically for livestock (e.g., AB Government, 2018; Černe et al., 2019; WDFW, 2013). Fewer resources are available for mortality and kill site assessment of wild ungulates, with some of the most extensive sources summarized in Table 1. Because these resources are comprehensive, our intent below is to highlight essential aspects that observers should pay attention to in the field, rather than an exhaustive treatment of the topic. By bringing mortality site investigation protocols into the spotlight, we wish to underline the importance of implementing field methodologies that are robust, repeatable, and that withstand rigorous peer review.

**TABLE 1:** Summary of some of the most comprehensive published resources available for identifying cause of mortality of wild ungulates, including the number of pages and photographs/illustrations dedicated to specific subjects

Resources	Region	Number of pages	Mortality and kill site assessment	Mammal track identification	Mammal scat identification	Bed identification	Other mammal signs
Alt and Eckert (2017)	US and Canada	120	66 pp., 33 visuals	4 pp., 17 visuals	9 pp., 41 visuals	–	–
Bang and Dahlstrøm (2001)	Europe	264	11 pp., 8 visuals	70 pp., 88 visuals	16 pp., 58 visuals	–	Yes
Elbroch and McFarland (2019)	US and Canada	680	56 pp. + 258 pp.,^a^ 87 visuals	99 pp., 912 visuals	51 pp., 248 visuals	Yes	Yes
Moskowitz (2010)	US and Canada, focus on Pacific northwest	364	8 visuals	239 pp.,^a^ 282 visuals	57 visuals	Yes	Yes
Nauta and Pot (2019)	Europe	445	–	445 pp., >1000 visuals	–	–	–
Rezendes (1999)	US and Canada, focus on northeast	336	4	275 pp.,^a^ 205 visuals	54	Yes	Yes
Rubines (2015)	Europe	239	6 pp., 2 visuals	27 pp., 66 visuals	18 pp., 67 visuals	–	Yes
Stonehouse et al. (2016)	Colorado and lower Rocky Mountains	80	61 pp.,^a^ 151 visuals	20 visuals	21 visuals		

^a^ Includes pages for all signs, as tracks, scats, etc., were mixed together in species accounts.

### 4.1 Equipment

We provide a list of equipment needed to conduct field investigations of mortality sites (Appendix S2: Table S1). While some equipment items are essential, others will only be needed if habitat data at the mortality site are collected. Such data have been shown to improve our understanding of habitat use and selection at small spatial scales (within foraging patches; Johnson, 1980). The use of field guides (e.g., Alt & Eckert, 2017; Elbroch & McFarland, 2019) is highly recommended. We also strongly encourage the collection of samples for DNA analysis that can elucidate the predator's identity in the laboratory (see Mumma et al., 2014 for an example). Many sampling methods allow for samples to be stored, should funding not be available to conduct analyses at the start of the fieldwork.

### 4.2 Safety

We recommend that mortality investigations be conducted by a pair of observers to meet safety requirements set by many universities and agencies. Bear spray should be carried in the hand when approaching potential kill, feeding or scavenging sites of large carnivores, including brown (*Ursus arctos*) and American black bears (*U. americanus*). Researchers should make noise as they approach suspected mortality sites, as well as periodically make noise during site and carcass investigations so that they do not surprise incoming large carnivores. Mortality site visits might need to be delayed in areas with a high density of brown bears, in order to minimize the risk to field crews.

### 4.3 Site investigations

Below we categorize mortality investigations into three sequential steps: Discovery, site analysis, and carcass analysis. We emphasize the importance of conducting site investigations as quickly as possible, as associated signs quickly disappear and are confused by accumulating sign of scavengers and decomposers.

#### 4.3.1 Discovery

The first step in any mortality investigation is to locate the collar signaling a mortality event. Neonate ungulates are typically fitted with expandable Very High Frequency (VHF) telemetry collars or ear tags, requiring the field crew to home in on the radio beacon using a handheld antenna attached to a receiver. Adult ungulates are increasingly fitted with satellite‐enabled GPS collars, which will transmit the mortality location. The horizontal error of GPS collars is typically less than 30 m (Tomkiewicz et al., 2010) and usually 5–10 m in our experience, depending upon terrain and vegetation.

When the signal strength from the telemetry collar indicates that the animal is nearby, extra care needs to be taken to avoid entering the mortality site by accident and potentially trampling important evidence. At this point, researchers should be aware of any possible evidence of predator or scavenger activity while attempting to find an area that allows them to observe the entire mortality site without disturbing it. From this location, take photographs of the entire site, inclusive of the entire carcass remains: First, document the animal ID digitally (e.g., by photographing the data sheet with the animal ID), and then photograph the general site characteristics, including the surrounding habitat. These photographs should capture first impressions of an area, including overall habitat and carcass disposition. Certain carnivores exhibit fine‐scale habitat selection to which they drag or carry the carcass (e.g., pumas and Eurasian lynx [*Lynx lynx*] often feed in thick cover); thus, the habitat in which the carcass is found offers insights into the potential predator. Prompt investigation can also identify human‐caused mortality such as roadkill or poaching by discovering clues such as blood spatter on the road surface and drag marks away from a road, and avoids the possibility that mortalities associated with humans could be erroneously attributed to predators.

#### 4.3.2 Site analysis

Once the general area has been documented with photographs and described on your data sheet, begin the site analysis. Below we highlight several signs researchers should actively look for in the field. Conducting the site investigation before investigating the carcass in detail minimizes the inadvertent destruction of signs that may be useful in determining cause of death.

If at any point in the investigation, you suspect poaching is the cause of death, stop the investigation immediately to avoid further site disturbance. Leave and contact local wildlife authorities.

##### 4.3.2.1 Drag marks

Prior to approaching the actual carcass, look for drag marks indicating the carcass has been moved. Pumas in northern California, for example, moved prey an average of 21.7 ± 4.3 m from the kill site to a feeding site that provided increased cover and potential concealment (Allen et al., 2015). While many GIS analyses are based on 30 m raster data, recording habitat variables at finer scales is important if study objectives include the description of habitat conditions at kill sites to assess prey vulnerability (Apps et al., 2013; Cristescu et al., 2014, 2019).

Drag marks are a linear depression in substrate, indicated by flattened vegetation with the tips of plants pointing in the direction the animal was dragged, displaced rocks moved in that same direction, and clumps of hair or substrate caught in logs, vegetation, and rock edges. By following the drag away from the carcass remains, investigators can backtrack to the site where the predator either killed the animal, or where it found the animal dead and dragged it to a more concealed location for consumption. Blood spatter, disturbed soil, and freshly broken or flattened vegetation with prey hair are signs of struggle and a predation event. Record the approximate size of the disturbed area associated with the kill site. Record whether there is a drag mark, and if yes, it can be useful to also record the length of the dragline (meters). Photograph the drag as well, along with various evidence of disturbances. Photographs taken at an angle sometimes provide clearer evidence than those taken directly from above, and having photographs from multiple angles and views is usually helpful. Disturbed areas, including the dragline itself, may expose soil, providing tracking substrate which might be the best location to look for footprints. Finding tracks, draglines, and disturbance indicative of a kill are often dependent on getting to the site quickly after the mortality.

##### 4.3.2.2 Tracks

Footprints provide evidence of the presence of predators and scavengers and may offer insights to the behaviors of predators, prey, and scavengers. Carry field guides during site investigations and photograph all footprints with a suitable scale (e.g., a ruler) so that they can be reviewed and assessed by others to aid in their identification. Ungulate tracks stomping the ground in the vicinity of neonate mortality sites may indicate a female defending her offspring or attempting to deter or distract the predator. Deep tracks of the ungulate and evidence of dragging feet can indicate a struggle.

Carnivores have differential probability of being recorded by researchers based on their morphology, weight, and track size. Large or social carnivores, such as bears (*Ursus* spp.) or wolves (*Canis lupus*), are more likely to be recorded than smaller predators. For example, a bear's tracks in soft moss register as depressions in the substrate, whereas small felid tracks may register little and be very difficult to distinguish. Tracks of chasing and/or pouncing predators (Appendix S3: Figure S1) may be visible near kill sites and indicate predation rather than scavenging.

Learning to differentiate between the different footprints of predators and scavengers takes years of experience but can be accelerated with support from field guides (Table 1) and training (e.g., www.trackercertification.com); nevertheless, imperfect tracks are often encountered in the field and can be difficult to interpret (Liebenberg, 1990). Tracking is easiest when the substrate is snow, soft soil, mud, or sand. However, fresh snow can cover tracks and other evidence, and extremely cold nights can freeze the surface of the snow, allowing light‐weight predators to walk aloft without leaving sign. Similarly, mud that dries up in the sun decreases the detection of carnivore tracks.

##### 4.3.2.3 Scat

Feces and their presentation may also betray predators and scavengers near a carcass (e.g., many felids form latrines and bury scats near kills). It is possible to identify the species that deposited scat with experience and training, but researchers often prove inconsistent in their ability to do so (e.g., Monterroso et al., 2019). Even the scats produced by an individual predator vary with diet, as well as the state and health of the animal. Field guides provide comprehensive resources for predator identification based on scat (Table 1), and increasingly online tools and resources may prove useful (e.g., the “Animals Don't Cover Their Tracks” Facebook group help in identifying signs from photographs).

A scat's relative freshness can be indicative of whether a predator and scavenger(s) were actually engaged with the carcass. A fresher scat is indicative of a carnivore that was actively involved with the carcass, whereas some scats might be older than when the kill was made and their presence at the site could be incidental. Fresh scat that is black in color likely includes blood, organs, and muscle tissue, which are the carcass parts typically consumed first, whereas scat that has lighter color and includes plenty of hair is indicative of a predator consuming less nutritious parts of the carcass once the preferred parts have been eaten. Scats with high blood content bleach white with age and exposure to the sun. The number of scats also provides a rough estimation of the time the animal spent at the site (e.g., brown bears defecate approximately four times per 24‐h cycle when consuming carcasses; Elfström et al., 2013), while varying sizes of scats from the same species may indicate if a female with young was present at the carcass.

##### 4.3.2.4 Hair

Ungulate hair caught in vegetation, logs, or rocks provides clues on their cause of death. If the hair is caught close to ground level along a drag, it indicates that the animal was likely dead or incapacitated and being dragged. Hair located high off the ground is indicative of a chase or struggle. Pumas, bobcats (*L. rufus*), and lynxes often pluck the hair of long‐haired prey, resulting in clumps of fur near the carcass and caught in vegetation (Appendix S3: Figure S2); this fur might also be accumulated as part of caching behavior (discussed below). If hair clumps include pieces of skin, then this may indicate that the animal was long dead when the fur was removed, rather than freshly killed.

Carnivore hair can provide important clues but is often inconspicuous at ungulate mortality sites. Search for carnivore hair on trails through vegetation leading to the carcass, as hair is often caught in broken vegetation, or on the resin or prickles of some plant species. Also, look for hair on low‐hanging branches directly above the carcass remains and in depressions that could be bed sites (see “Bed sites” section below). Collecting carnivore hair enables identification of the predator and scavenger species in the laboratory, by analyzing cuticula scales and cross‐sections with a microscope. The Alaska Fur ID Project (https://alaskafurid.wordpress.com/), Teerink (2004) and Tóth (2017) are resources to identify hair to species level.

##### 4.3.2.5 Bed sites

Flattened impressions or sometimes excavated depressions may indicate places where predators or scavengers rested near the carcass. For example, bears often spend time resting at ungulate carcasses (Video S1) and their bed sites are particularly easy to determine in the field. Characteristics of the resting areas for various species in temperate regions are described in Elbroch and McFarland (2019).

##### 4.3.2.6 Rubs, scrapes, and scratches

Predators and scavengers may scrape the ground or rub and scratch nearby logs and tree trunks as part of marking and cleaning behaviors. Felids, for example, sometimes scratch tree trunks or create scrapes in the duff layer with their hind feet near their kill sites (White et al., 2011). Male carnivores scent mark more often than females (e.g., Allen et al., 2014; Krofel et al., 2017) and may also mark carcasses more often, so to avoid bias do not rely only on marking signs to make inferences on carnivore individuals at the mortality site. The details of some of these signs can be found in the guides to interpreting field evidence (Table 1).

#### 4.3.3 Carcass analysis

First, estimate the number of days the animal has been dead, based upon the date the animal's beacon was last heard alive, and the date the collar switched to a mortality signal. Note whether there were any circumstances that hindered an immediate search for the carcass.

**FIGURE 1:**
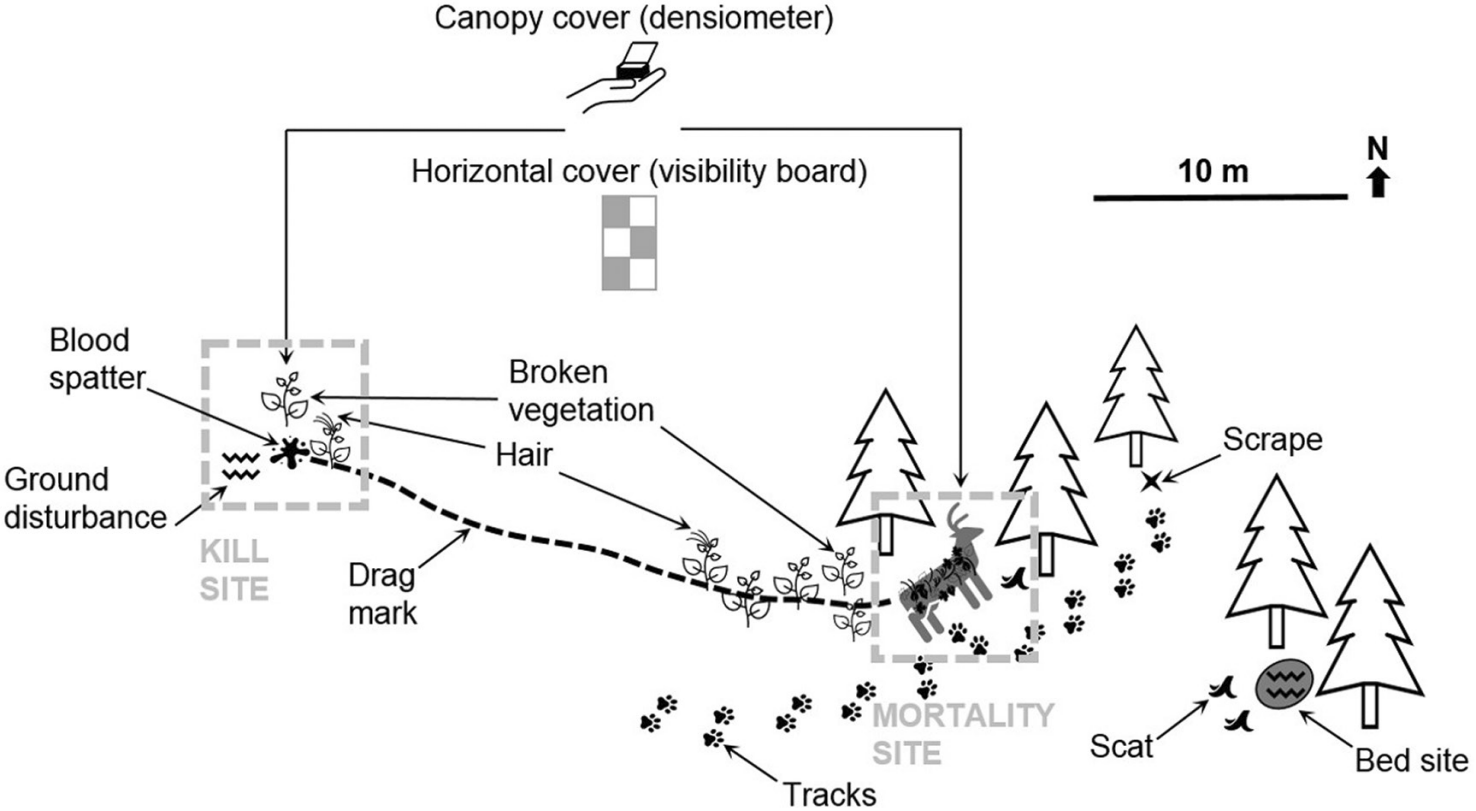
Conceptual figure for aspects of ungulate mortality site assessment in studies on marked ungulates. The carcass is surrounded by supplementary sign (e.g., tracks, scat, beds, drag marks, and blood spatter) that can help identify the predator and may be cached with materials such as leaves, twigs, and duff. Habitat measurements (canopy cover and horizontal cover) can also provide clues on the predator(s) present at the kill and may be used to evaluate the role of habitat in predation risk

Carcass analysis includes external and internal investigation via necropsy. When you are ready to investigate the carcass remains, wear gloves. If only bones or bone fragments remain of the carcass, measure the total length and width of the area containing bones (Figure 1). The relationship between carcass size and the size of the disturbed area (small, medium, and large) can be used as one of the first indications of the carnivore involved. Below is a list of topics of interest that should be recorded.

##### 4.3.3.1 Cached and buried remains

Note whether the carcass is visible and unadorned, or whether there is evidence of a cache pile or a burial site. Felids and some ursids (e.g., Allen et al., 2021b) sometimes cache ungulate carcasses at ground level with debris collected from the area surrounding the carcass (Video S2). Canids more often bury parts of the carcass in often highly camouflaged holes below ground level they dig with their front paws. When material such as forest floor duff is available, cache sites are often neatly piled (Appendix S3: Figure S3). For larger prey or when the materials available for caching are in short supply, sometimes only the exposed parts of the carcass such as the thoracic cavity opened to access organs are cached with grass clumps or twigs. In winter, the carcass can be cached with snow, but carcasses might not be cached as often as in summer (Appendix S3: Figure S4). Pumas and Eurasian lynx separate the gut pile from the rest of the carcass, and they may also cache it independently.

Some canids, like coyotes (*C. latrans*), often split neonate carcasses into two and bury the different ends, and often cache neonate heads and necks (Appendix S3: Figure S5). Wolves will also cache food, particularly in the summer when they are hunting in smaller groups, and typically cache regurgitated food chunks rather than carcass parts (Petersen & Ciucci, 2003). These burial sites differ in several aspects from cache sites of felids. Canids camouflage their burial sites, which are difficult to identify without the aid of the VHF transmitter on prey (Appendix S3: Figure S6). Burial sites can be located far from the kill site; in one instance, we found that a coyote buried a mule deer neonate >1 km from the kill site, while wolves have been reported to cache food up to 5 km from kills and rarely make buried caches near their kill (Petersen & Ciucci, 2003). In contrast, felid caches are typically <100 m from the kill site and rarely >200 meters away.

##### 4.3.3.2 State and placement of the carcass

Record direct signs in the immediate vicinity of the carcass, including the presence of blood as well as the carcass position. For example, fresh kills yield blood whereas opening an old carcass yields little or none. Pumas and Eurasian lynx neatly open carcasses, and often there is little pooled blood on the ground, whereas canids slash prey that bleeds from numerous openings leaving more sign on the ground. Also record the openings into the carcass, how many there are and their location. Coyotes, for example, often enter ungulate carcasses from the rear, whereas pumas and wolves enter from the point where the stomach touches a hindquarter. If the carcass has not been completely consumed, describing the parts that have been consumed (organs, rump, legs, intestines, brain, and bones) can provide valuable clues. In general, solitary predators open carcasses in one place, whereas canids and other social carnivores separate carcasses into parts across larger areas. See resources in Table 1. Although it can provide clues on the predators that were present at the site, carcass consumption patterns should be treated as a secondary identifier that is paired with more distinctive, species‐specific carnivore sign, such as tracks, DNA, or hair. Perhaps future assessments of species‐specific carnivore handling of ungulate carcasses in experimental settings could improve observer confidence in investigating ungulate mortality based on carcass consumption patterns.

If the carcass is relatively intact, record the position of the legs. Legs tucked under the body are often indicative of death by malnutrition or disease, whereas legs to the side are often indicative of an animal that was predated or that struggled as it died (e.g., due to poisons or injury).

Record the percentage of the carcass that has been consumed. The size of the carnivore responsible will determine its intake rate and satiation, and therefore, you can use the state of the carcass and the time since the ungulate died to begin to speculate on the size of the carnivore or scavenger responsible. While a bear may consume a young fawn entirely in one sitting, a bobcat will take much longer and the fawn carcass will be largely intact if the mortality site is investigated promptly. Social carnivores and scavengers will also consume carcasses faster than solitary individuals (e.g., wolf packs consume large prey faster than a single puma). When prey are abundant, predators may kill and feed little or not at all (Kruuk, 1972). Conversely, a predator that makes frequent returns to feed, such as a solitary felid returning to a cache site, may complete several feeding sessions before the transmitter remains still for long enough to log a mortality. Avian scavengers in large numbers will often deplete the carcass before the investigator arrives. Because of these potential influences on carcass condition, inferences derived from field inspection of the carcass state should be interpreted cautiously and always in conjunction with additional site evidence.

##### 4.3.3.3 Necropsy and carcass details

Field staff should be trained in conducting necropsies safely, or they should be required to remove carcasses in their entirety so that someone with training can conduct the necropsy. Wherever and whenever possible, state veterinary laboratories or equivalent facilities should be used for necropsies. Even with training on how to perform field necropsies, field researchers typically lack the formal extensive training and experience that wildlife veterinarians have with identifying factors that could indicate the cause of death, particularly non‐predation‐related mortalities. Samples can then be sent to external laboratories if necessary for disease testing, to ensure the accuracy of results. We caution against conducting necropsies in the field without the use of personal protective equipment, due to risks of disease transmission (Wong et al., 2009) and physical injury.

Before the necropsy, sample for pathogens. Consult your local agency veterinarian for potential pathogens in your study area. Swab the nasal cavity or the mouth for bacteria that are associated with pneumonia (Cassirer et al., 2017) and tuberculosis (Barasona et al., 2017). The recent development of molecular methods provides opportunities to collect DNA as well, which in combination with results from the mortality site investigations, can confirm the presence of specific predators (see Mumma et al., 2014 for a general description of DNA protocols). If hair different than that of the prey is found, it should also be collected for DNA analysis. Containers for DNA swabs must be uniquely labeled with sufficient information to link the sample back to a specific mortality investigation (date, animal ID, GPS coordinates, type of sample, etc.).

Record evidence of bite wounds and claw marks to throat, back of the neck, face, muzzle, skull, back, rump, and rear and front legs. Swab areas around bite marks, tooth punctures including bones, or where fur has been matted from saliva to collect DNA to identify predator species (Mumma et al., 2014). Quite commonly bite and claw marks will not be visible on the hide, so skinning the carcass should be carried out to locate wounds penetrating the skin and/or signs of hemorrhaging near bite marks or bruising (Appendix S3: Figure S7). Hematoma indicates blood clots, which form upon impact or bite when the animal is still alive. Measure the distance between bite marks with calipers to match with dental patterns of predators (Elbroch & McFarland, 2019). The tooth puncture diameters are also useful to measure as these measurements can help identify the species responsible. Be careful not to stretch the skin while making measurements, skewing their accuracy.

Record whether the trachea is intact, being careful not to puncture it while skinning the carcass; canids may bite prey multiple times on the face, neck, skull, hind legs, and other parts of the body (Bowns, 1995; Mech, 1970). Felids generally deliver one clean bite to the throat (adult large ungulates), or back of the neck or skull (small adult or neonate ungulates; Murphy & Ruth, 2010; Sunquist & Sunquist, 2002), and it is sometimes possible to see the four punctures corresponding to the four canines. Felids also sometimes kill adult large ungulates by enclosing the muzzle of the prey in their mouth and subsequent suffocation (Kitchener et al., 2010; Leyhausen & Tonkin, 1979).

Record the state of long bones, including the potential presence of chew marks, and whether any are missing. Some predators lack the strength to crack adult ungulate long bones, or do not eat them. Long bones also provide information about the nutritional condition of the prey. A rough indication of the nutritional condition can be taken in the field by assessing the color and consistency of bone marrow (e.g., Hornocker, 1970). However, we recommend researchers collect entire, unbroken bones for a more accurate investigation of marrow fat content in the laboratory (Lamoureux et al., 2011).

An animal in poor body condition would have depleted many of its fat reserves and might be more likely to die from malnutrition or disease before being discovered and killed by a predator. The marrow of an animal that used many of its fat reserves is red, pink, or spotted in color (Appendix S3: Figure S8). Also, liver or gelatinous consistency marrow are clear signs of an animal in poor body condition. White, yellow, and/or solid marrow does not necessarily indicate that the animal was in good overall condition, but simply that the condition was better (on a broad gradient from substantially to marginally so) than individuals with other marrow phenotypes (Mech & Delgiudice, 1985). Long bones (femur or humerus) or standardized sections thereof can be collected, labeled, and transferred to the lab for bone marrow analysis. Note that the bones should be frozen as soon as possible. In addition, the time interval between site investigation and analysis of marrow should be as short as possible to avoid possible deterioration of the marrow. The marrow's aspect is not reliable for estimating body condition of neonate ungulates, as most will have little fat reserves and high vascularization in the marrow due to the growth process, resulting in naturally red marrow color.

In addition, investigate the equipment used to mark the ungulates, including ear tags and collars. These might have blood stains and other potential signs of carnivores (both predators and scavengers) on them, including puncture marks, tears, and chewing. Sometimes only the collar and/or ear tag(s) are found, and investigators must assess site evidence to identify whether the marked ungulate is dead or simply lost its mark. From our experience, the collar and ear tag are typically near each other at bear and bobcat kills of neonate ungulates, indicating a concentrated feeding site.

#### 4.3.4 Habitat analysis

While the habitat at the mortality site may differ across carnivore species (e.g., Apps et al., 2013), we advise against relying on habitat features to identify the predator that made the kill. In general, sites where felids and bears consume prey are concealed, whereas canids appear to consume ungulates in more open and flatter areas (May et al., 2008). However, large carcasses cannot always be dragged to concealed locations and some will be consumed at the kill site. Scavengers, too, may drag carcasses for considerable distances. Investigators should rely on physical evidence left by the predator to establish the species that made the kill, thereby avoiding habitat‐related confirmation bias and allowing inferences on predation risk by habitat type.

Depending on study objectives, the final step of the mortality site investigation could involve a detailed description and measurement of habitat attributes. This could be important because conditions at fine scales may differ from data available from GIS layers that often lack the detailed information necessary to evaluate the role of habitat in predator‐specific mortalities. If habitat features are also recorded at random sites with no evidence of carcasses, then habitat selection for kill and feeding sites (fourth‐order selection, Johnson, 1980) can be analyzed in a used–unused design (e.g., Cristescu et al., 2014).

Important habitat variables to measure are context‐dependent and tailored to specific objectives, but should include abiotic recordings (slope and aspect), a description of the dominant tree and shrub communities, as well as the presence of snow (which can affect the detection of predator sign). To evaluate potential links between predation and cover as a key element in predator–prey interactions (Gorini et al., 2012), we advise measuring vertical (canopy) cover using a densiometer, as well as horizontal cover using boards for visibility assessment (Figure 1).

## 5 CAUSE OF MORTALITY CASE STUDY FOR MULE DEER

From 2015 to 2020, we conducted a study aimed at determining the roles of top‐down and bottom‐up effects on the population dynamics of mule deer in northern California. Similar to many other ungulate studies, our objectives were to quantify vital rates required to estimate population growth and thus included rates and causes of neonate and adult mortality. The multi‐predator community capable of killing mule deer in our study area was composed of puma, black bear, coyote, and bobcat. Large raptors were present, but we never identified an avian predator as the cause of deer mortality although this is known to happen (Gilbert, 2016).

In June–July of 2017–2019 and a brief pilot period in 2016, we captured mule deer neonates by hand and with salmon nets. We searched for neonates with spotlights during the birthing season while driving along an extensive network of paved and unpaved roads during the night. When we spotlighted female deer that behaved suspiciously (e.g., hesitant to move away), we performed quick searches for neonates bedded in their vicinity. We also captured neonates we encountered during daylight fieldwork activities. We fitted all neonates with expandable VHF radio collars (VECTRONIC Aerospace). All capture and handling procedures were permitted (University of California Santa Cruz IACUC WILMC1509 and WILMC1811; California Department of Fish and Wildlife Scientific Collecting permit SC‐10859). We monitored neonates on a daily basis for the first 3 months and on a weekly basis thereafter up to 12 months of age. Such changes in monitoring intensity are not unusual as the larger sizes of older neonates facilitate mortality investigations. We visited mortality sites as soon as possible, typically on the same day that we heard collars on mortality beacon. In some instances, however, it took several days or even weeks to relocate telemetered neonates, resulting in delayed mortality site investigations.

As part of our study, we also captured adult female deer and fitted them with GPS collars equipped with mortality sensors and satellite transmission capability (VECTRONIC Aerospace). We programmed collars to send a mortality notification via e‐mail if the collar was stationary for >4 consecutive hours. We attempted to visit adult mortality sites within 24 h of e‐mail notification by navigating to the location cluster using a handheld GPS. In practice, we sometimes received e‐mails days after the mortality event occurred due to predators and scavengers moving the collar while feeding or because collars were buried obstructing satellite access. Because of these external factors, some collars never sent satellite mortality notifications and these mortalities were only discovered during regular ground‐based VHF monitoring several days or weeks after occurrence.

**FIGURE 2:**
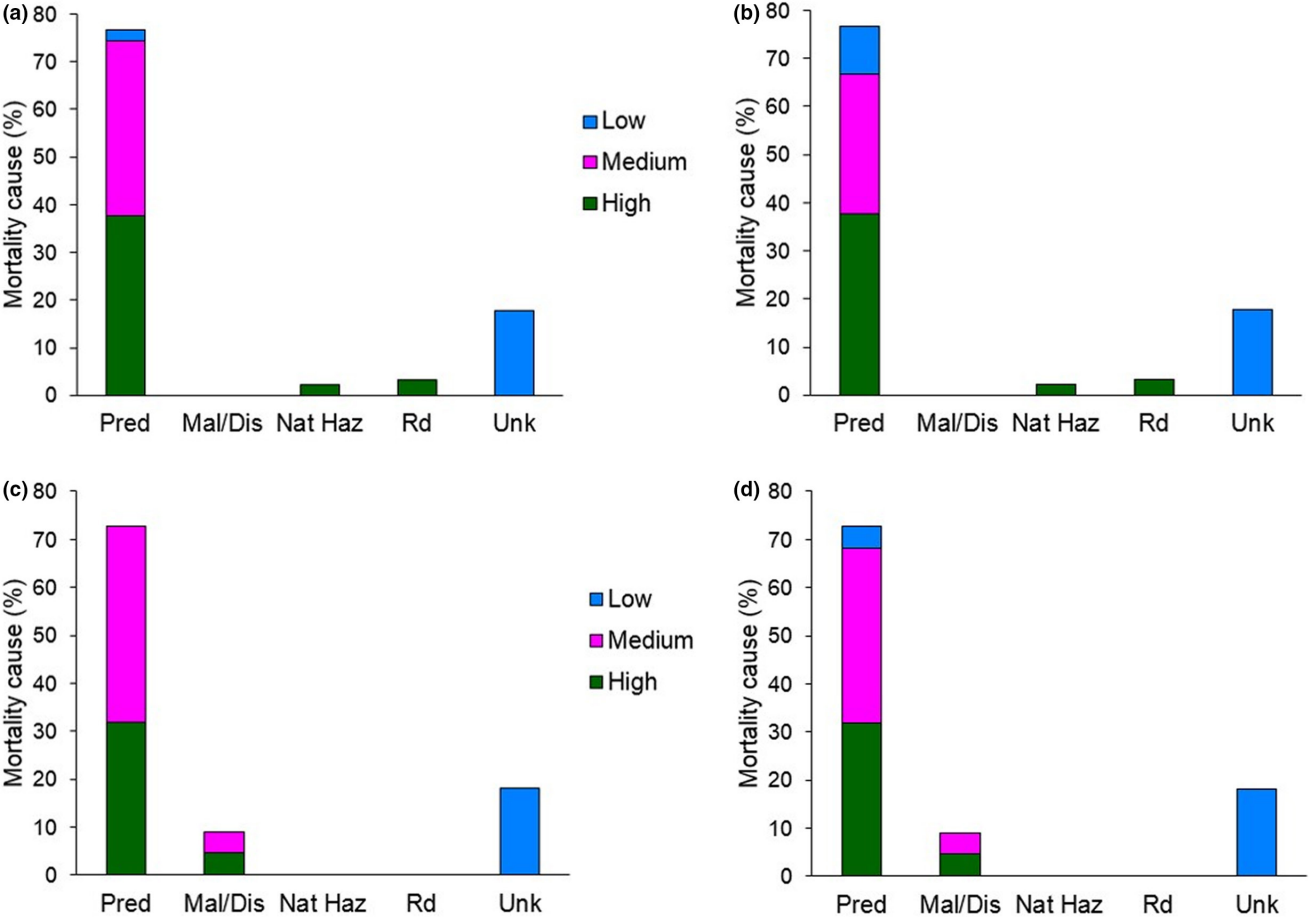
Mortality cause for marked mule deer neonates <1 year (*n* = 90; (a) predator species pooled, (b) predator species‐specific cause of mortality) and deer >1 year (*n*_yearlings_ = 2, *n*_adults_ = 20; (c) predator species pooled, (d) predator species‐specific cause of mortality) in northern California (2016–2020). Mortality data are presented along with confidence level in cause of death assignment (Low, Medium, and High). Two additional recorded mortalities are excluded due to extensive time elapsed between mortality and field site investigation (one fawn: >1 month; one adult deer: >3 months). Earlier data on adult mortality (2015; *n* = 5) were collected opportunistically and are therefore also not included in the graphs. Pred—predation, Mal/Dis—malnutrition/disease, Nat Haz—natural hazard, Rd—roadkill, Unk—unknown

All field personnel were trained theoretically and then accompanied in the field by an experienced investigator to learn and practice our mortality site investigation protocol. The theoretical training occurred before the first day in the field and involved going through the protocol step‐by‐step, conceptualizing the layout and assessment procedure for a typical site, and discussing different scenarios that could be encountered. Practical training occurred with oversight from experienced investigators, who provided guidance on approach to the site, search patterns, and type of evidence sought. Personnel were only permitted to carry out site assessments independently based on positive feedback from experienced investigators accompanying them in the field and after the field crew leader was comfortable with their performance usually after carrying out multiple site investigations together. Mortalities and their potential causes (Figure 2) were discussed, and photographs reviewed once back from the field. All mortality investigation datasheets and accompanying photographic evidence were scrutinized by the field crew leader, who periodically reviewed case findings with an experienced investigator.

**FIGURE 3:**
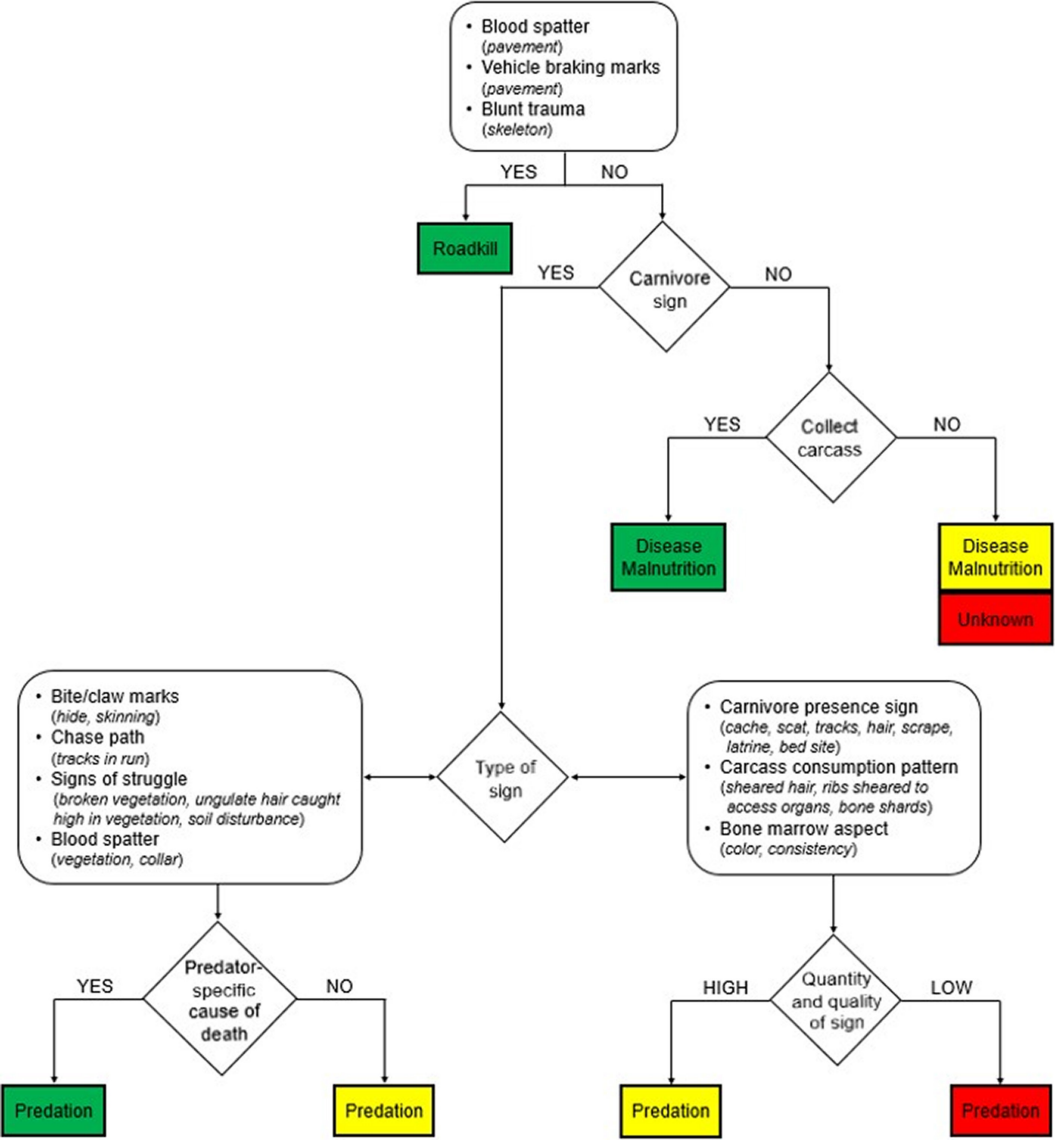
Confidence level assignment for ungulate cause of death on a study on mule deer survival in northern California (2016–2020). Mortality causes were assigned with High (green), Medium (yellow) or Low confidence (red). The flow chart can be applied to other systems but requires that field investigations of ungulate mortality sites are prompt, especially in systems with complex carnivore guilds and/or high predator and scavenger abundances

Once all data were collated, we assigned each mortality assessment a relative confidence level (High, Medium, Low) based on evidence present at the site (Figure 3). Unknown causes of mortality were included by default in the Low confidence level category. Most variability in confidence over cause of death assignment was related to predation (Figure 2). The majority of causes of mortality were attributed with High or Medium confidence. However, if High confidence in mortality assessment is the desired outcome (i.e., Medium and Low confidence events are discarded), then the percentage of known mortality causes decreases substantially (approximately 60% for both neonates and adults in our study; neonates: Appendix S4: Table S1; adult females: Appendix S4: Table S2).

Over the duration of the study, we captured 145 neonates, 91 of which were confirmed dead during their first year of life (Data S4). Without confidence level assignment, predation accounted for 77% of mortalities recorded (*n*_coyote_ = 21, *n*_bear_ = 17, *n*_puma_ = 14, *n*_bobcat_ = 9, *n*_predator species not identified_ = 9). The remaining mortalities were due to roadkill (*n* = 3), natural hazards (*n* = 2), and unknown causes (*n* = 16). When only High confidence data were included, predation accounted for 37% of confirmed mortalities.

We captured a total of 86 adult females, 4 of which died of capture‐related mortality. Of the remaining 82 deer, 26 were confirmed dead at the end of fieldwork in July 2020 (Data S4). Five mortalities occurred in 2015, when mortality site visitations were opportunistic and detailed records of mortality site visits by agency personnel were not available. These mortalities were, therefore, excluded from the cause of mortality analysis. However, two of the collared neonates that survived their first year of life and died as yearlings were included for analyses with the adult females. Without confidence level assignment, predation accounted for 70% of the mortalities recorded (*n*_puma_ = 10, *n*_coyote_ = 5, *n*_predator not identified_ = 1). The remaining mortalities were due to malnutrition (*n* = 2) and unknown causes (*n* = 5). When only High confidence data were included, predation accounted for only 30% of mortalities.

We used logistic regression to test whether the time elapsed between the animal mortality and the day of the site investigation (elapsed time = independent continuous variable) influenced our ability to assign mortalities to a particular cause. We coded mortalities as the dependent binary variable with successful identification of cause of death irrespective of confidence level (1) versus mortalities of unknown cause (0). The date of mortality was based on carcass freshness and other field evidence and within the bounds of when the animal's VHF beacon was last heard alive and when it was first heard on mortality mode. When field evidence was inconclusive, we set the mortality date as the midpoint between the day when the collar was heard on mortality mode and when it was last heard alive. Because we did not expect to be able to identify cause of death when lengthy time had elapsed, we excluded data for one fawn that had died >1 month before the site visit and for one adult female that had died >3 months prior to our site visitation.

**FIGURE 4:**
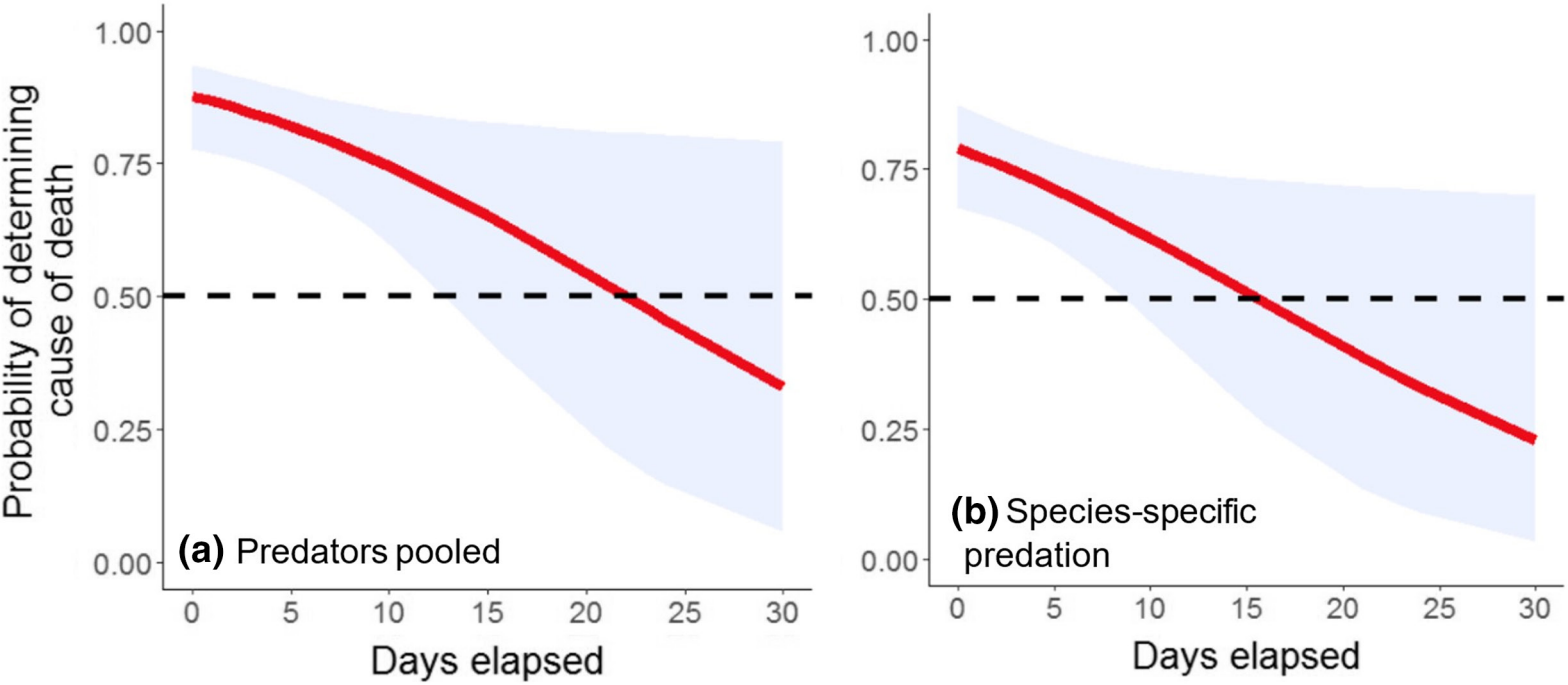
Probability of identifying cause of death of mule deer neonates as a function of number of days elapsed between mortality and site visitation by field crews, when predation records are pooled (a) or differentiated among predator species (b). 95% confidence levels are presented in gray shading. Data are for radiocollared neonates confirmed dead (*n* = 90) in northern California (2016–2020) and comprise mostly predation events (*n* = 69), but also roadkill (*n* = 3) and natural hazard deaths (*n* = 2), as well as unknown causes of mortality (*n* = 16). The horizontal dashed line indicates the probability threshold (*p* = .50) for discriminating cause of death as per field procedures and conditions, which is not the same as probability that the discrimination was correct

Based on cause of death recorded as predation (pooled across predator species), roadkills, and natural hazards, we found that increasing the time interval between mortality and site visitation impeded our ability to identify cause of death for neonates, but the relationship was weak (β_Days elapsed_ = −0.089, SE = 0.041, *p* = .029; deviance explained = 0.056; Figure 4a). We found a similar pattern when using data on cause of death recorded as predator species‐specific predation, along with roadkills and natural hazards (β_Days elapsed_ = −0.085, SE = 0.040, *p* = .035; deviance explained = 0.045; Figure 4b). Overall, the probability of identifying predator species‐specific predation events in the field was lower and required prompter site visitation than when cause of death was pooled across predator species (Figure 4). For deer >1 year old, the number of days elapsed did not significantly influence our ability to assign cause of death for either predation pooled across predator species (β_Days elapsed_ = −0.029, SE = 0.050, *p* = .565; deviance explained = 0.015) or species‐specific predation (β_Days elapsed_ = −0.015, SE = 0.048, *p* = .749; deviance explained = 0.004). Mortality site investigations for neonates, therefore, appear most sensitive to timing of field visitation and mortality cause is more difficult to determine for neonates as time progresses, even though investigations of neonate mortality sites occurred faster (mean ± SD, 4.0 ± 5.7 days) compared to visiting mortalities of deer >1 year old (8.3 ± 10.4 days).

**FIGURE 5:**
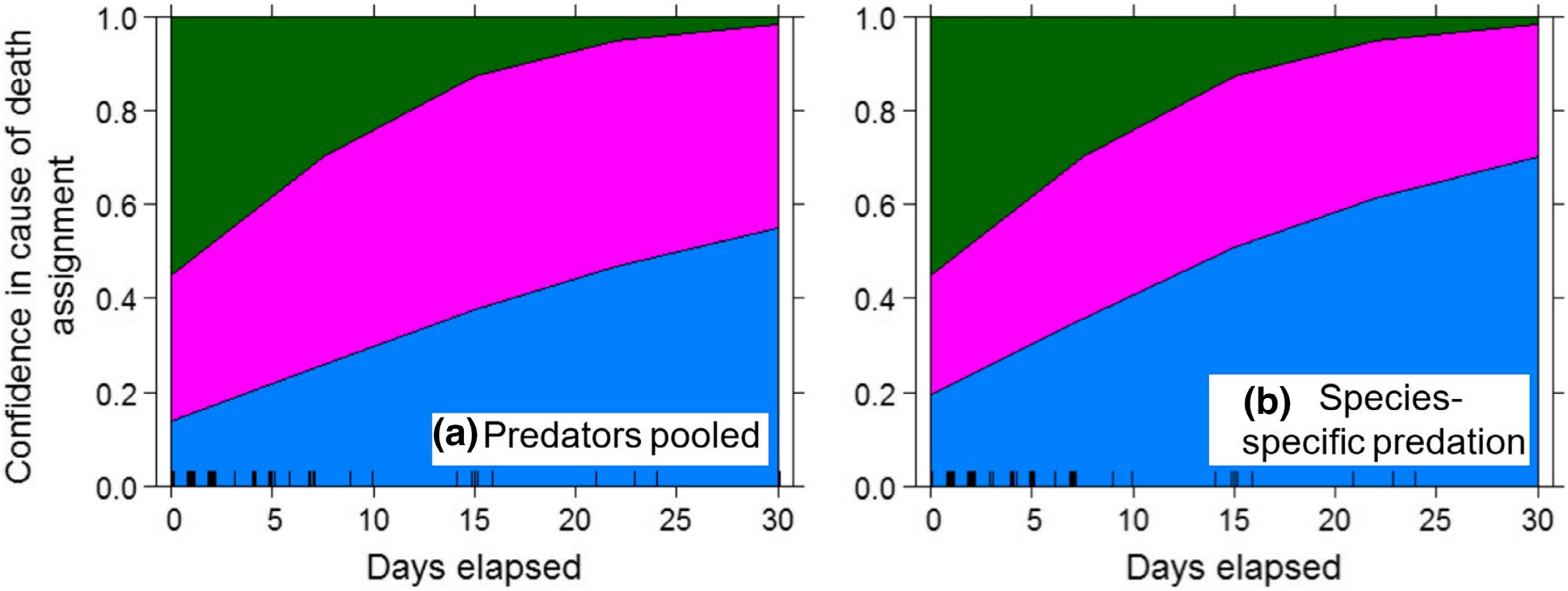
Probability of confidence level for identifying cause of death of mule deer neonates as a function of number of days elapsed between mortality and site visitation by field crews. Data are presented for predation records pooled (a) or differentiated among predator species (b). Confidence levels in cause of death assignment were High (green), Medium (purple), or Low (blue). Data are for radiocollared neonates confirmed dead (*n* = 90) in northern California (2016–2020) and comprise mostly predation events (*n* = 69), but also roadkill (*n* = 3) and natural hazard deaths (*n* = 2), as well as unknown causes of mortality (*n* = 16)

We used multinomial regression to understand how outcomes of field visitations varied with regard to confidence level in cause of death assignment, based on the number of days elapsed between the mortality event and the site visit. The dependent multinomial variable had three levels: Low (reference category), Medium, or High confidence in mortality cause assessment, whereas elapsed time was the independent variable. The days that elapsed before we conducted site investigations did not influence our classification of mortality with Medium confidence when compared to Low confidence assignments. However, as days elapsed, we were less likely to classify neonate mortalities with High confidence (β_Days elapsed [High confidence]_ = −0.164, SE = 0.070, *p* = .019; Figure 5a). Similarly, as days elapsed, the probability of assigning High confidence dropped (β_Days elapsed [High confidence]_ = −0.161, SE = 0.068, *p* = .018; Figure 5b) for predator species‐specific predation on neonates and non‐predation‐related mortality. Overall, the confidence levels in identifying predator species‐specific predation events in the field appeared lower than when cause of death was pooled across predator species (Figure 5). For deer >1 year old, time elapsed between mortality and site visit was not significantly associated with confidence level assigned from field investigation for either predation pooled across predator species (β_Days elapsed (Medium confidence)_ = −0.027, SE = 0.054, *p* = .620; β_Days elapsed (High confidence)_ = −0.031, SE = 0.057, *p* = .587) or species‐specific predation (β_Days elapsed (Medium confidence)_ = −0.011, SE = 0.052, *p* = .829; β_Days elapsed (High confidence)_ = −0.020, SE = 0.055, *p* = .718). Taken together, both of the analyses provide evidence that neonate mortality site assessments are more influenced by the time between mortality and site visit than site assessments for older deer.

One key reason for neonate mortality site assessments being more sensitive to timing of investigations than mortalities of adult deer is the size of the carcass. Neonate carcasses are smaller, and predators and scavengers can consume them rapidly, often almost entirely, especially in the neonates' first months of life. Allowing time to pass before site visitation obscures field evidence from consumption and hinders mortality identification efforts. In contrast, carcasses of deer that are >1 year old are substantially larger and, therefore, take longer to consume. Site evidence is also more likely to accumulate because of the size of the animal. For example, a drag mark is more easily detected for a large‐bodied carcass than a small neonate that some predators can carry without leaving drags. Small carcasses can be disarticulated easily and, therefore, pieces can be taken away by scavengers with little sign left, whereas large carcasses are more difficult to move and have more surface area and volume available to register sign of predation.

Another factor which could affect the outcomes of neonate mortality site assessments is the predator and scavenger community that feeds on the carcass. We assessed the presence of predators and scavengers at carcasses, relying on species‐specific sign such as tracks, scat, caches, bite, and claw marks. Based on the evidence collected in the field, up to three predators capable of killing deer in our study area were present at the same carcass, although most often we only found sign of one predator species at a given carcass (82% for neonates, 78% for does). The presence of >1 predator at a carcass was independent of whether the carcass was a neonate or adult (Pearson's Chi‐squared test, Χ^2^ = 0.211, df = 1, *p* = .646). However, the number of predators we documented from sign could possibly be lower than what we would have been able to document had we monitored the same carcasses with camera traps (i.e., Allen et al., 2021a). The presence of multiple predators at the same mortality site not only highlights the importance of carrying out site visitations promptly but also for reviewers and editors to require a full description of mortality assessment protocols prior to publishing cause of death information. This ensures transparency in reporting and would enable readers to assess the reliability of the data and conclusions.

The importance of rapid visitation of mortality sites for small‐bodied prey such as neonates is illustrated by the following examples in which we located neonates prior to scavenging obscuring field sign. On one occasion, we located a telemetered neonate that had been killed by a bobcat. During a site revisit the following day, we found that all bobcat sign had disappeared, as a bear had visited the site, destroyed the subtler signs of bobcat and carried the carcass >200 m away. We found the collar and several bear scats at the new location, which was more concealed than the site where the bobcat had first killed the neonate. Were we to visit the mortality site within 48 h instead of within 24 h, available evidence would have led us to erroneously classify the cause of death as bear instead of bobcat. On two other occasions, we discovered two completely intact neonates, which we transferred to the Wildlife Investigations laboratory of the California Department of Fish and Wildlife for necropsy. The first (time elapsed between mortality and site visitation = 1 day) died from a sharp stick that had penetrated the chest. The second fawn (time elapsed = 2 days) became stuck in a pile of logs and died of head injuries and possibly exposure trying to free itself. Were we to visit these sites later, we might have been unable to find intact carcasses and mortality causes would have remained unknown, or in the absence of adequate training, they might have been attributed to predators.

Scavengers can obscure or confuse signs of predators at adult ungulate carcasses as well. For example, bears and wolves push pumas off their kills (Allen et al., 2021a; Elbroch & Kusler, 2018), and if the investigators arrive after this occurs, they may misclassify the predator. Even though the number of days elapsed since mortalities did not significantly influence the confidence levels of observers in assigning cause of death for deer >1 year old, we encourage prompt site visitation for adult ungulates also. Our site visitation was relatively rapid for adults (8.3 ± 10.4 days, if one abnormal observation with 169 days elapsed is omitted), which might have contributed to our ability to identify cause of death. In addition, the predator guild in our study system was simplified, with brown bears, wolves, and wolverines (*Gulo gulo*) being historically present, but absent during the study period. The densities of carnivores in our study system might also be lower than in other areas, and carnivore and predation sign can quickly become hard to detect at mortality sites of both neonate and adult ungulates if the sites are not investigated rapidly. Prompt site investigations are particularly important in ecosystems with diverse guilds of carnivores that occur at high abundance, as other carnivores can utilize carcasses and obscure the evidence needed to determine cause of death (e.g., predation vs. other), and the predator responsible for the kill. The disappearance of field sign can also be compounded by rainfall or fresh snow, emphasizing the need for rapid mortality site visitation across ungulate age and size classes.

The flow chart (Figure 3) derived for our study is intended as a starting point to help orient researchers and managers on how to conduct their own mortality site investigations. The particulars of other study areas may require some site‐specific adjustment. For example, predator and scavenger diversity and density in different areas will affect the evidence at the mortality site and on the carcass, and the size of the ungulate species will influence carcass state (e.g., neonates of large‐bodied ungulates will afford more opportunities for predation sign to be preserved on the carcass and be recorded by field crews than deer neonates will). Irrespective of the study system, cause of death assignments should be interpreted with caution in the Discussion sections of papers reporting cause‐specific mortalities, especially for young neonates as it can be particularly difficult to differentiate predation from scavenging for this age group.

We have included the blank datasheet used in our own mortality site investigations, as supplementary material in MS Word format for easy editing (Appendix S5). We recommend a comments section which can be used for text as well as for sketching the mortality site, thereby facilitating recollection of important facts. The sketch could include type of animal sign and its distribution, as well as possibly topographic, water, vegetation features, and a scale. Upon request from the editor and reviewers, these detailed records should be shared along with photographs, thereby ensuring quality control. Increasingly, journals require authors to make their programming code and raw data available to readers. We argue that field data that are used for descriptive and statistical analyses should also undergo a review. Field data are the backbone on which codes are run and we emphasize the need for high standards of data collection.

Table 2 shows the information that we recommend be reported whenever mortality of marked ungulates needs to be summarized in survival and cause of mortality studies. Information to be made public should include the last time an individual was located alive, the first time it was heard on mortality as well as the date the mortality site investigation was conducted. Cause of mortality and confidence level in mortality assignment should also be indicated. We invite researchers and practitioners to consider using the table templates that we provide herein for tagged ungulate mortality site visits. We also propose that representative photographs of the kill site (general area and carcass) be preserved and made available to readers upon request. We provide examples for coyote (Appendix S3: Figure S9), black bear (Appendix S3: Figure S10), bobcat (Appendix S3: Figure S11), and puma (Appendix S3: Figure S12) predation on mule deer fawns, as well as puma predation on adult mule deer (Appendix S3: Figure S13).

**TABLE 2:** Individual‐level data that should be reported for ungulate mortality site investigations

Deer ID	Age class	Sex	Age (estimated)	Weight at capture (kg)	Body condition at capture	Capture date	Collar type	Last date observed alive	Date VHF Beacon heard on mortality	Mortality date collar	Date mortality retrieved	Status
R080	Neonate	Male	Neonate	2.8	Fair	24‐Jun‐18	Vectronic VHF Fawn Expandable	31‐Jul‐18	2‐Aug‐18	1‐Aug‐18^a^	2‐Aug‐18	Dead; Predation (*Coyote*)
R075	Neonate	Female	Neonate	4.7	Good	14‐Jun‐18	Vectronic VHF Fawn Expandable	24‐Nov‐18	29‐Nov‐18	26‐Nov‐18^a^	29‐Nov‐18	Dead; Unknown
410	Adult	Female	3 years	47.6	Good	8‐Mar‐18	Vectronic Survey Globalstar	21‐Mar‐18	NA^b^	22‐Mar‐18^c^	22‐Mar‐18	Dead; Predation (*Puma*)
203	Adult	Female	4 years	56.2	Good	15‐Jul‐16	Vectronic Vertex Plus Iridium	4‐Oct‐18	6‐Oct‐16	5‐Oct‐18^d^	6‐Oct‐16	Dead; Unknown

*Note*: Data for two neonate and two adult mule deer that were confirmed dead in northern California are provided as example. The examples illustrate some of the opportunities as well as difficulties encountered in cause of death identification even when field visitation of mortality sites is prompt, as well as the challenges to obtain the mortality date. Investigators should report how mortality dates were estimated and must not hesitate to record animal status as “Unknown” when confronted with substantial uncertainty.

^a^ Mortality date set at halfway between the last date the neonate was located alive and the date its VHF transmitter was heard on mortality mode.

^b^ Mortality notification received via e‐mail from the satellite collar.

^c^ Mortality date corresponded to the mortality status recorded by the satellite collar.

^d^ The satellite collar failed to send a mortality notification; therefore, the mortality date was set at halfway between the last date the animal was located alive and the date its VHF transmitter was heard on mortality mode.

## 6 CONCLUSIONS AND FUTURE WORK

Identifying the cause of mortality in the field remains a critical foundation for many questions related to wildlife, population and conservation ecology, as well as predator–prey interactions. Cause of mortality data have been used to inform predator control programs, but evidence on whether such strategies were justified or had the desired effects is often not compelling (Bergstrom et al., 2014; Clark & Hebblewhite, 2021; Treves, 2009; Woodroffe & Redpath, 2015). One aspect that will undoubtably contribute to the credibility of quantitative assessments in survival and population dynamics investigations is the standardization of methodology for mortality site investigations. We thus encourage investigators to describe field procedures in greater detail in the Methods section or as Supplementary material and to refer to comprehensive sources consulted for their mortality site investigations (Table 1). We have also highlighted benefits for using a qualitative scale for ranking confidence level in cause of death assignment. As part of our proposed framework, we also propose researchers maintain a database of project‐specific datasheets including links to photographic evidence used to ascertain cause of mortality. Making this evidence available upon request during the review process and publishing key supportive evidence as an Appendix to journal articles should become the standard of the publication process. Such a framework will greatly improve transparency, assist reviewers in assessing the results, and ultimately facilitate standardization and more credible comparisons of cause of mortality across studies.

Prompt site visitation is critical for mortality site assessments, especially in systems with complex carnivore guilds. We recognize that rapid site investigation is a common challenge in field studies, especially in remote and rugged settings and is also affected, among other factors, by the size of the field team and number of animals monitored. We encourage authors to be transparent on the temporal aspects of site visitation as well as the challenges they encountered in their study. Researchers should not be hesitant in reporting the inability to determine cause of death, and journals should not automatically reject articles based on this issue, but the reasoning for not identifying cause of mortality should be stated.

Assigning the cause of death in mortality site investigations is particularly challenging for multi‐predator systems that also have a complex community of scavengers. We caution that even in single predator systems, the cause of death should not automatically default to the a priori (anticipated) predator, as many large carnivores scavenge (e.g., Knopff et al., 2010). Assessment of cause of death should instead rely on evidence at the site as well as on the carcass itself, such as tracks, scat, caching or burial, bite, and claw marks. Erroneous assignment of predation and misidentification of the predator are pernicious errors because standard study designs have no means of estimating the magnitude of their effects. Thus, studies of cause‐specific mortality can be sensitive to the prevalence of what we term Medium confidence assignments. In the face of uncertainty, clearly articulating the standard of evidence applied in generating these cause‐specific assignments is key to maintaining transparency in research.

Carcass necropsy in the laboratory and by a veterinarian as well as collection of DNA samples to identify predator species are useful practices to account for field uncertainty and should be employed more frequently. In some situations, detailed necropsies and DNA evidence not only can eliminate the uncertainty associated with the predator species responsible for the kill, but via DNA analysis can also pinpoint individuals repeatedly involved in predation. This is particularly important when the prey or predator species are of conservation concern and may eliminate the need for blanket predator control (Ernest et al., 2002; Mumma et al., 2014).

Statistical methods, such as data augmentation within a Bayesian hierarchical framework incorporating expert knowledge, have been proposed to refine cause of mortality assessments and to account for the inherent uncertainty in any data collected by multiple observers with different skills and experiences (Walsh et al., 2018). Such approaches also provide field researchers greater opportunity to report a lack of confidence in identifying the predator responsible, or even whether it was a predation event. Given that most ungulates will be affected by interactions between top‐down and bottom‐up effects (Hopcraft et al., 2010), developing models that can incorporate multiple sources of information (e.g., mortality site information, DNA sampling, and GPS data from predator collars) while also incorporating multiple sources of uncertainty will be an important tool to identify the impact of predation on the population dynamics of ungulates. Transparent and thorough mortality site investigations with estimates of error will be critical for developing these models.

We also encourage manufacturers of wildlife tracking equipment to further experiment with developing GPS units for ungulate neonates (data loggers on expandable collars) that are capable of transmitting mortality notifications remotely, while ensuring that animal welfare standards are met. Data from GPS units can be used to identify additional areas to search for cause of death sign, which can be particularly helpful when collars are carried off away from kill sites or cache locations. An additional temperature logger or advanced 3‐axis accelerometers incorporated in ungulate collars could possibly record activity signatures indicative of kills vs. scavenging events. To our knowledge, accelerometers have not been used for this purpose in ungulate collars, although they see increasing applications in wildlife ecology research (Wilmers et al., 2015).

Conservation and management actions based on findings from mortality site investigations may include habitat modification and predator population manipulation, which is increasingly controversial. Mortality assessment data must therefore be high quality, credible and the data collection process transparent and repeatable. We hope that the layout of procedural steps herein could assist ecologists, managers, and especially early career scientists to devise research protocols, perform fieldwork, and ensure accountability.

## AUTHOR CONTRIBUTIONS

**Bogdan Cristescu:** Data curation (lead); formal analysis (lead); investigation (equal); methodology (equal); project administration (equal); writing – original draft (lead). **L. Mark Elbroch:** Investigation (equal); methodology (equal); resources (supporting); writing – review and editing (equal). **Tavis D. Forrester:** Investigation (equal); methodology (equal); writing – review and editing (equal). **Maximilian L. Allen:** Investigation (equal); methodology (equal); writing – review and editing (equal). **Derek B. Spitz:** Investigation (equal); methodology (equal); writing – review and editing (equal). **Christopher C. Wilmers:** Funding acquisition (lead); investigation (equal); methodology (equal); project administration (lead); resources (lead); supervision (lead); writing – review and editing (equal). **Heiko U. Wittmer:** Conceptualization (lead); funding acquisition (lead); investigation (equal); methodology (equal); project administration (lead); resources (lead); supervision (lead); writing – review and editing (equal).

## CONFLICT OF INTEREST

The authors have no conflicts of interest to declare.

## Supporting information


Appendix S1

Appendix S2

Appendix S3

Appendix S4

Appendix S5

Video S1

Video S2

## Data Availability

Data associated with this study is available at Dryad (https://doi.org/10.7291/D1GD50) and includes 3 datasets (2 .csv and 1 .xls) representing the results of mortality site investigations for collared mule deer in northern California. R code files (2 .R) are also included.
