# Supplementary material for: Standardizing protocols for determining the cause of mortality in wildlife studies
Source: Ecol Evol. 2022 Jun 23;12(6):e9034. doi: 10.1002/ece3.9034 (PMC9219102; doi:10.1002/ece3.9034)
Supplement: Supplementary file 1 — Appendix S1 [file ECE3-12-e9034-s001.docx]

**Appendix S1**

Cristescu, B., L. M. Elbroch, T. D. Forrester, M. L. Allen, D. B. Spitz, C. C. Wilmers, and H. U. Wittmer. Standardizing protocols for determining the cause of mortality in wildlife studies. Ecology and Evolution.

Table S1. Key parameters extracted from studies on survival of marked neonate ungulates in North America and Europe. A total of 25 peer-reviewed studies were randomly selected from the peer-reviewed literature, based on searches in Google Scholar.

| **Ungulate species** | **Ungulate age (class)** | **Study area** | **Predator community^1^** | **Protocol description (# words)^2^** | **In-text citations to protocol**  **(# references)^3^** | **Lab necropsy^4^** | **Monitoring (days)^5^** | **Predator DNA^6^** | **Reference** |
| --- | --- | --- | --- | --- | --- | --- | --- | --- | --- |
| Bighorn sheep | Lamb | South Dakota | Multiple | 22 | 0 | Yes | Daily (60 days post-capture)  3–4 times/week thereafter | No | Smith et al. 2014 |
| Black-tailed deer | Fawn | California | Multiple | 28 | 0 | No | Daily (capture to mid-Sep) Every 10-14 days thereafter | No | Marescot et al. 2015 |
| Caribou | Calf | Québec | Multiple | 52 | 0 | No | ≥ 3 days (up to Jul 19)  ≥ 2 weeks (until late Aug)  Less frequently thereafter | No | Pinard et al. 2012 |
| Caribou | Calf | Newfoundland | Multiple | 161 | 2 | Yes | Daily (7 days post‐capture)  Every 2–4 days (following 4–5 weeks)  Every 5–10 days (until Aug)  Fortnightly or monthly (thereafter) | Yes | Mahoney et al. 2015 |
| Elk | Calf | Idaho | Multiple | 85 | 2 | Yes | ≥ 3 days/week (first 8–10 weeks)  Weekly (Aug – Sep)  1–3 times/month (thereafter until the  following Jun) | Yes | White et al. 2010 |
| Elk | Calf | Pennsylvania | Multiple | 24 | 0 | Yes | Daily (capture to July 31)  Weekly (thereafter) | No | DeVivo et al. 2011 |
| Elk | Calf | North Carolina, Tennessee | Unspecified | 33 | 2 | No | 3 times/day (first 2 weeks of life)  ≥ 1/day (thereafter up to 5 weeks) | No | Yarkovich et al. 2011 |
| Elk | Calf | Montana | Multiple | 111 | 1 | No | Daily (capture to mid-Jul)  3 times/week (mid-Jul to late Aug)  2–3 times/week (Sep to May) | Yes | Eacker et al. 2016 |
| Moose | Calf | Alaska | Multiple | 21 | 2 | No | Daily (until mid-Jun)  Every other day (until early Jul)  Every 5 days (until mid-Aug)  Every other week (until Nov)  Thereafter once per month | Yes | Keech et al. 2011 |
| Moose | Calf | Ontario | Multiple | 48 | 1 | Yes | Daily (first month of life) Twice a week (rest of summer)  Once a week (rest of first year of life) | No | Patterson et al. 2013 |
| Moose | Calf | Minnesota | Multiple | 304 | 3 | Yes | Daily | Yes | Severud et al. 2015 |
| Moose | Calf | Minnesota | Multiple | 114 | 2 | No | Unspecified | Yes | Obermoller et al. 2019 |
| Mule deer | Fawn | Washington | Unspecified | 37 | 1 | Yes | Every other day (fawn capture to Sep 12) | No | Johnstone-Yellin et al. 2009 |
| Mule deer | Fawn | Arizona | Multiple | 135 | 0 | Yes | ≥ 1/day (capture to Fall)  1/month (thereafter until fawns were 1 year of age) | No | Quintana et al. 2016 |
| Mule deer | Fawn | New Mexico | Unspecified | 265 | 3 | Yes | ≥ 1/week | No | Lomas & Bender 2007 |
| Pronghorn | Neonate | Montana, Wyoming | Multiple | 59 | 1 | No | Daily (until Aug 1) | No | Barnowe-Meyer et al. 2009 |
| Pronghorn | Neonate | South Dakota | Multiple | Unspecified | 1 | Yes | 2-3 times/day (until Aug 31) | No | Jacques et al. 2015 |
| Reindeer | Calf | Finland | Multiple | 90 | 3 | Yes | 2-3 day intervals (summer until the end of Aug)  1/week (Sep-Oct) | No | Nieminen et al. 2011 |
| Roe deer | Fawn | Norway | Multiple | 34 | 0 | Yes | Daily (until 2 months of age)  10 times/month (until 1 month of age)  5 times/month (from 1 to 2 months of age) | No | Panzacchi et al. 2008 |
| Roe deer | Fawn | Germany | Single^7^ | 21 | 0 | No | 1-3/week or more frequent | No | Heurich et al. 2012 |
| White-tailed deer | Fawn | South Carolina | Multiple | 401 | 7 | No | Daily (first month)  3–4 times/week (thereafter) | No | McCoy et al. 2013 |
| White-tailed deer | Fawn | Michigan | Multiple | 66 | 4 | No | Daily up to 5 times/week | No | Duquette et al. 2014 |
| White-tailed deer | Fawn | Louisiana | Multiple | 355 | 9 | Yes | Every 8 hours (until 6 weeks of age)  Once daily (until 12 weeks of age) | Yes | Shuman et al. 2017 |
| White-tailed deer | Fawn | Pennsylvania | Multiple | 54 | 1 | Yes | Twice daily (capture to mid-Aug)  1–7 times weekly (mid-Aug to early Dec)  1–3 times weekly (thereafter) | No | Gingery et al. 2018 |
| White-tailed deer | Fawn | Michigan | Multiple | 479 | 6 | No | 1–3 times/day (capture to Aug 31); other ones monitored every 2-4 days  Weekly (Sep-Dec) | No | Kautz et al. 2019 |

^1^ Predator community as described in the study area description of the Methods section

^2^ Number of words in the Methods section describing the protocol for mortality site investigation, with a focus on field analyses of the site, carcass, habitat, and comments on field-derived cause of death assignment. Transmitter monitoring regime, carcass discovery, carcass pickup for lab necropsy, and sample collection and processing are not included in the word count. Some studies included references to literature/protocol which contributed to the word count

^3^ Number of in-text citations in the Methods section that reference literature on mortality site investigation procedures/protocol

^4^ Lab necropsy included on a case-by-case basis

^5^ Frequency of monitoring the study animals based on telemetry tags

^6^ Predator DNA was typically swab, sometimes hair or scat

^7^ Single predator community inferred but not explicitly stated

Table S2. Key parameters extracted from studies on survival of marked adult ungulates in North America and Europe. A total of 25 peer-reviewed studies were randomly selected from the peer-reviewed literature, based on searches in Google Scholar.

| **Ungulate species** | **Ungulate age (class)^1^** | **Study area** | **Predator community^2^** | **Protocol description (# words)^3^** | **In-text citations to protocol**  **(# references)^4^** | **Lab necropsy^5^** | **Monitoring (days)^6^** | **Predator DNA^7^** | **Reference** |
| --- | --- | --- | --- | --- | --- | --- | --- | --- | --- |
| Bighorn sheep | Adult | California | Single^8^ | 57 | 0 | No | ≥ Twice monthly | No | Johnson et al. 2013 |
| Bighorn sheep | Adult | South Dakota | Multiple | 36 | 0 | Yes | Daily (lambing season through 60 days postpartum)  1-3 times/week thereafter | No | Smith et al. 2015 |
| Bighorn sheep | Adult | Wyoming | Unspecified | Unspecified | 0 | No | Once monthly (winter) More frequently (summer) | No | Courtemanch et al. 2017 |
| Black-tailed deer | Adult | California | Multiple | 28 | 0 | No | ≥ Twice weekly (summer) Every 10-14 days thereafter | No | Marescot et al. 2015 |
| Caribou | Adult | British Columbia | Multiple | 129 | 0 | No | 1 flight/2 weeks | No | Apps et al. 2013 |
| Caribou | Adult | Québec | Unspecified | Predation assumed | 0 | No | NA | No | Losier et al. 2015 |
| Elk | Adult | Washington | Unspecified | 110 | 0 | No | Twice/month (Dec–Jul)  2–3 flights/week (Aug–  Nov) | No | McCorquodale et al. 2011 |
| Elk | Adult | Colorado; New Mexico | Multiple | Unspecified | 0 | No | Every 2–4 weeks | No | Webb et al. 2011 |
| Elk | Adult | North Carolina, Tennessee | Unspecified | 33^9^ | 2 | Yes | 2 times/week | No | Yarkovich et al. 2011 |
| Elk | Adult | Colorado | Unspecified | 150 | 1 | Yes | ≥ 1/week | No | Monello et al. 2014 |
| Elk | Adult | Kentucky | Unspecified | Unspecified | 0 | Yes | Weekly or bi-weekly (mid-Feb-Jul 31)  3 times/week (Aug 1-mid-Feb)  2 times/week (males) | No | Slabach et al. 2018 |
| Moose | Adult | Minnesota | Unspecified | 74 | 1 | Yes | Weekly | No | Lenarz et al. 2009 |
| Moose | Adult | Alaska | Multiple | 42 | 2 | No | Daily (May and early Jun)  Monthly (thereafter) | No | Keech et al. 2011 |
| Mountain goat | Adult | Alaska | Multiple | Unspecified | 0 | No | ≥ 1/month; often multiple times/month | No | White et al. 2011 |
| Mule deer | Adult | New Mexico | Multiple | 53 | 1 | Yes | ≥1 times/week | No | Bender et al. 2012 |
| Mule deer | Adult | Arizona | Multiple | 135^9^ | 0 | Yes | Unspecified | No | Quintana et al. 2016 |
| Mule deer | Adult | Washington | Multiple | 73 | 2 | No | Daily | No | Dellinger et al. 2018 |
| Mule deer | Adult | Oregon | Multiple | 227 | 2 | No | Unspecified | No | Schuyler et al. 2019 |
| Pronghorn | Adult | Montana, Wyoming | Multiple | 59^9^ | 1 | No | Twice weekly (spring, summer, and autumn) | No | Barnowe-Meyer et al. 2009 |
| Pronghorn | Adult | North Dakota | Multiple | 145 | 3 | No | Once every 10 days | No | Kolar et al. 2012 |
| Pronghorn | Adult | South Dakota | Multiple | 118 | 4 | No | 3–5 times/week (Jan-Aug)  1 time/week (Nov-Jan, Sep-Nov) | No | Keller et al. 2013 |
| Roe deer | Adult | Germany | Unspecified | 21 | 0 | No | 1-3 times/week or more frequent | No | Heurich et al. 2012 |
| Roe deer | Adult | Poland | Multiple | 294 | 0 | No | 10 times per month | No | Sönnichsen et al. 2017 |
| White-tailed deer | Adult | Washington | Multiple | 73 | 2 | No | Daily | No | Dellinger et al. 2018 |
| White-tailed deer | Adult | Kentucky | Unspecified | 137 | 0 | Yes | Daily (first four weeks post-capture)  Weekly (thereafter) | No | Haymes et al. 2018 |

^1^ Adult age class pools adult, subadult and yearling

^2^ Predator community as described in the study area description of the Methods section

^3^ Number of words in the Methods section describing the protocol for mortality site investigation, with a focus on field analyses of the site, carcass, habitat, and comments on field-derived cause of death assignment. Transmitter monitoring regime, carcass discovery, carcass pickup for lab necropsy, and sample collection and processing are not included in the word count. Some studies included references to literature/protocol which contributed to the word count

^4^ Number of in-text citations in the Methods section that reference literature on mortality site investigation procedures/protocol

^5^ Lab necropsy included on a case-by-case basis

^6^ Frequency of monitoring the study animals based on telemetry tags

^7^ No swab, hair or scat were collected

^8^ Single predator community inferred but not explicitly stated

^9^ Mortality site investigation procedures for adult ungulates not clearly specified, therefore the word count reported herein was adopted from the neonate protocol

**Literature reviewed**

Apps, C. D., B. N. Mclellan, T. A. Kinley, R. Serrouya, D. R. Seip, and H. U. Wittmer. 2013. Spatial factors related to mortality and population decline of endangered mountain caribou. Journal of Wildlife Management 77:1409–1419.

Barnowe-Meyer, K. K., P. J. White, T. L. Davis, and J. A. Byers. 2009. Predator-specific mortality of pronghorn on Yellowstone's northern range. Western North American Naturalist 69:186–194.

Bender, L. C., B. D. Hoenes, and C. L. Rodden. Factors influencing survival of desert mule deer in the greater San Andres Mountains, New Mexico. Human–Wildlife Interactions 6:245–260.

Courtemanch, A. B., M. J. Kauffman, S. Kilpatrick, and S. R. Dewey. 2017. Alternative foraging strategies enable a mountain ungulate to persist after migration loss. Ecosphere 8:e01855.

Dellinger, J. A., C. R. Shores, M. Marsh, M. R. Heithaus, W. J. Ripple, and A. J. Wirsing. 2018. Impacts of recolonizing gray wolves (*Canis lupus*) on survival and mortality in two sympatric ungulates. Canadian Journal of Zoology 96:760–768.

DeVivo, M. T., W. O. Cottrell, J. M. DeBerti, J. E. Duchamp, L. M. Heffernan, J. D. Kougher, and J. L. Larkin. 2011. Survival and cause-specific mortality of elk *Cervus canadensis* calves in a predator rich environment. Wildlife Biology 17:156–165.

Duquette, J. F., J. L. Belant, N. J. Svoboda, D. E. Beyer Jr., and P. E. Lederle. 2014. Effects of maternal nutrition, resource use and multi-predator risk on neonatal white-tailed deer survival. PLoS ONE 9:e100841.

Eacker, D. R., M. Hebblewhite, K. M. Proffitt, B. S. Jimenez, M. S. Mitchell, and H. S. Robinson. 2016. Annual elk calf survival in a multiple carnivore system. Journal of Wildlife Management 80:1345–1359.

Gingery, T. M., D. R. Diefenbach, B. D. Wallingford, and C. S. Rosenberry. 2018. Landscape-level patterns in fawn survival across North America. Journal of Wildlife Management 82:1003–1013.

Haymes, C. A., J. R. McDermott, G. S. W. Jenkins, W. E. Bowling, J. T. Hast, K. L. Johannsen, and J. J. Cox. 2018. Survival and cause-specific mortality of white-tailed deer in Southeastern Kentucky. Journal of the Southeastern Association of Fish and Wildlife Agencies 5:90–96.

Heurich, M., L. Möst, G. Schauberger, H. Reulen, P. Sustr, and T. Hothorn. 2012. Survival and causes of death of European Roe Deer before and after Eurasian Lynx reintroduction in the Bavarian Forest National Park. European Journal of Wildlife Research 58:567–578.

Jacques, C. N., J. A. Jenks, T. W. Grovenburg, and R. W. Klaver. 2015. Influence of habitat and intrinsic characteristics on survival of neonatal pronghorn. PLoS ONE 10:e0144026.

Johnson, H. E., M. Hebblewhite, T. R. Stephenson, D. W. German, B. M. Pierce, and V. C. Bleich. 2013. Evaluating apparent competition in limiting the recovery of an endangered ungulate. Oecologia 171:295–307.

Johnstone-Yellin, T. L., L. A. Shipley, W. L. Myers, and H. S. Robinson. 2009. To twin or not to twin? Trade-offs in litter size and fawn survival in mule deer. Journal of Mammalogy 90:453–460.

Kautz, T. M., J. L. Belant, D. E. Beyer Jr., B. K. Strickland, T. R. Petroelje, and R. Sollmann. 2019. Predator densities and white‐tailed deer fawn survival. Journal of Wildlife Management 83:1261–1270.

Keech, M. A., M. S. Lindberg, R. D. Boertje, P. Valkenburg, B. D. Taras, T. A. Boudreau, and K. B. Beckmen. 2011. Effects of predator treatments, individual traits, and environment on moose survival in Alaska. Journal of Wildlife Management 75:1361–1380.

Keller, B. J., J. J. Millspaugh, C. Lehman, G. Brundige, and T. W. Mong. 2013. Adult pronghorn (*Antilocapra americana*) survival and cause-specific mortality in Custer State Park, S.D. The American Midland Naturalist 170:311–322.

Kolar, J. L., J. J. Millspaugh, T. W. Mong, and B. A. Stillings. 2012. Survival and cause-specific mortality of pronghorn in Southwestern North Dakota. The American Midland Naturalist 167:164–173.

Lenarz, M. S., M. E. Nelson, M. W. Schrage, and A. J. Edwards. 2009. Temperature mediated moose survival in Northeastern Minnesota. Journal of Wildlife Management 73:503–510.

Lomas, L. A., and L. C. Bender. 2007. Survival and cause-specific mortality of neonatal mule deer fawns, North-Central New Mexico. Journal of Wildlife Management 71:884–894.

Losier, C. L., S. Couturier, M.-H. St-Laurent, P. Drapeau, C. Dussault, T. Rudolph, V. Brodeur, J. A. Merkle, and D. Fortin. 2015. Adjustments in habitat selection to changing availability induce fitness costs for a threatened ungulate. Journal of Applied Ecology 52:496–504.

Mahoney, S. P., K. P. Lewis, J. N. Weir, S. F. Morrison, J. G. Luther, J. A. Schaefer, D. Pouliot, and R. Latifovic. 2016. Woodland caribou calf mortality in Newfoundland: insights into the role of climate, predation and population density over three decades of study. Population Ecology 58:91–103.

Marescot, L., T. D. Forrester, D. S. Casady, and H. U. Wittmer. 2015. Using multistate capture–mark–recapture models to quantify effects of predation on age-specific survival and population growth in black-tailed deer. Population Ecology 57:185–197.

McCorquodale, S. M., P. A. Wik, and P. E. Fowler. 2011. Elk survival and mortality causes in the Blue Mountains of Washington. Journal of Wildlife Management 75:897–904.

McCoy, J. C., S. S. Ditchkoff, J. B. Raglin, B. A. Collier, and C. Ruth. 2013. Factors influencing survival of white-tailed deer fawns in coastal South Carolina. Journal of Fish and Wildlife Management 4:280–289.

Monello, R. J., J. G. Powers, N. T. Hobbs, T. R. Spraker, M. K. Watry, and M. A. Wild. 2014. Survival and population growth of a free-ranging elk population with a long history of exposure to Chronic Wasting Disease. Journal of Wildlife Management 78:214–223.

Nieminen, M., H. Norberg, and V. Maijala. 2011. Mortality and survival of semi-domesticated reindeer (*Rangifer tarandus tarandus* L.) calves in northern Finland. Rangifer 31:71–84.

Obermoller, T. R., G. D. Delgiudice, and W. J. Severud. 2019. Maternal behavior indicates survival and cause‐specific mortality of moose calves. Journal of Wildlife Management 83:790–800.

Panzacchi, M., J. D. C. Linnell, J. Odden, M. Odden, and R. Andersen. 2008. When a generalist becomes a specialist: patterns of red fox predation on roe deer fawns under contrasting conditions. Canadian Journal of Zoology 86:116–126.

Patterson, B., J. F. Benson, K. Middel, K. Mills, A. Silver, and M. Obbard. 2013. Moose calf mortality in Central Ontario, Canada. Journal of Wildlife Management 77:832–841.

Pinard, V., C. Dussault, J.-P. Ouellet, D. Fortin, R. Courtois. 2012. Calving rate, calf survival rate, and habitat selection of forest-dwelling caribou in a highly managed landscape. Journal of Wildlife Management 76:189–199.

Quintana, N. T., W. B. Ballard, M. C. Wallace, P. R. Krausman, J. de Vos Jr., O. Alcumbrac, C. A. Cariappa, and C. O’Brien. 2016. Survival of desert mule deer fawns in Central Arizona. The Southwestern Naturalist 61:93–100.

Schuyler, E. M., K. M. Dugger, and D. H. Jackson. 2019. Effects of distribution, behavior, and climate on mule deer survival. Journal of Wildlife Management 83:89–99.

Severud, W. J., G. Del Giudice, T. R. Obermoller, T. A. Enright, R. G. Wright, and J. D. Forester. 2015. Using GPS collars to determine parturition and cause-specific mortality of moose calves. Wildlife Society Bulletin 39:616–625.

Shuman, R. M., M. J. Cherry, T. N. Simoneaux, E. A. Dutoit, J. C. Kilgo, M. J. Chamberlain, and K. V. Miller. 2017. Survival of white-tailed deer neonates in Louisiana. Journal of Wildlife Management 81:834–845.

Slabach, B. L., J. T. Hast, S. M. Murphy, W. E. Bowling, R. D. Crank, G. Jenkins, K. L. Johannsen, and J. J. Cox. 2018. Survival and cause-specific mortality of elk *Cervus canadensis* in Kentucky, USA. Wildlife Biology 2018: wlb.00459.

Smith, J. B., J. A. Jenks, T. W. Grovenburg, and R. W. Klaver. 2014. Disease and predation: sorting out causes of a bighorn sheep (*Ovis canadensis*) decline. PLoS ONE 9:e88271.

Smith, J. B., and T. W. Grovenburg. 2015. Survival of female bighorn sheep (*Ovis canadensis*) in the Black Hills, South Dakota. The American Midland Naturalist 174:290–301.

Sönnichsen, L., T. Borowik, T. Podgórski, K. Plis, A. Berger, and B. Jędrzejewska. 2017. Survival rates and causes of mortality of roe deer *Capreolus capreolus* in a rural landscape, eastern Poland. Mammal Research 62:141–147.

Webb, S. L., M. R. Dzialak, J. J. Wondzell, S. M. Harju, L. D. Hayden-Wing, and J. B. Winstead. 2011. Survival and cause-specific mortality of female Rocky Mountain elk exposed to human activity. Population Ecology 53:483–493.

White, C. G., P. Zager, and M. W. Gratson. 2010. Influence of predator harvest, biological factors, and landscape on elk calf survival in Idaho. Journal of Wildlife Management 74:355–369.

White, K. S., G. W. Pendleton, D. Crowley, H. J. Griese, K. J. Hundertmark, T. McDonough, L. Nichols, M. Robus, C. A. Smith, and J. W. Schoen. 2011. Mountain goat survival in Coastal Alaska: effects of age, sex, and climate. Journal of Wildlife Management 75:1731–1744.

Yarkovich, J., J. D. Clark, and J. L. Murrow. 2011. Effects of black bear relocation on elk calf recruitment at Great Smoky Mountains National Park. Journal of Wildlife Management 75:1145–1154.
