# Supplementary material for: Standardizing protocols for determining the cause of mortality in wildlife studies
Source: Ecol Evol. 2022 Jun 23;12(6):e9034. doi: 10.1002/ece3.9034 (PMC9219102; doi:10.1002/ece3.9034)
Supplement: Supplementary file 2 — Appendix S2 [file ECE3-12-e9034-s002.docx]

**Appendix S2**

Cristescu, B., L. M. Elbroch, T. D. Forrester, M. L. Allen, D. B. Spitz, C. C. Wilmers, and H. U. Wittmer. Standardizing protocols for determining the cause of mortality in wildlife studies. Ecology and Evolution.

Table S1. Equipment needed for mortality site (including the collection of possible DNA samples) and associated habitat investigations.

| **Description (alphabetical)** | **Quantity** | **Category** | **Purpose** |
| --- | --- | --- | --- |
| Backpack | 1 | M & H | Equipment carrying |
| Callipers | 1 | M | Inter-canine distances (P), puncture diameters (P) |
| Camera | 1 | M & H | Photo recording |
| Clinometer | 1 | H | Slope |
| Clipboard | 1 | M & H | Data recording |
| Compass | 1 | H | Aspect |
| Cover board | 1 | H | Horizontal cover |
| Data sheets (regular & waterproof paper) | 1 | M & H | Data recording |
| Densiometer | 1 | H | Vertical cover |
| Field guide (plants) | 1 | H | Species identification (V) |
| Field guide (tracks & sign) | 1 | M | Species identification (P) |
| Field notebook | 1 | M & H | Data recording |
| Glove sets (disposable) | 2 | M | Carcass handling (p) |
| GPS handheld | 1 | M & H | Navigation |
| Hand sanitizer | 1 | M | Hygiene |
| Hand saw | 1 | M | Bone marrow access (p) |
| Knife | 2 | M | Multipurpose |
| Magnifying glass | 1 | H | Species identification (V) |
| Measuring tape (20 m) | 1 | H | Measurement standardization (V) |
| Paper envelopes | 4 | M | Hair collection (P), Tooth collection for age (p) |
| Pencil | 2 | M & H | Data recording |
| Pliers | 1 | M | Tooth extraction for age (p) |
| Swabs for DNA analysis | 4 | M | Species identification (P) |
| Ziploc bag for garbage | 1 | M | Used glove disposal |

M – Mortality; H – Habitat; P – predator; p – prey; V – vegetation
