# Supplementary material for: Standardizing protocols for determining the cause of mortality in wildlife studies
Source: Ecol Evol. 2022 Jun 23;12(6):e9034. doi: 10.1002/ece3.9034 (PMC9219102; doi:10.1002/ece3.9034)
Supplement: Supplementary file 3 — Appendix S3 [file ECE3-12-e9034-s005.docx]

**Appendix S3**

Cristescu, B., L. M. Elbroch, T. D. Forrester, M. L. Allen, D. B. Spitz, C. C. Wilmers, and H. U. Wittmer. Standardizing protocols for determining the cause of mortality in wildlife studies. Ecology and Evolution.


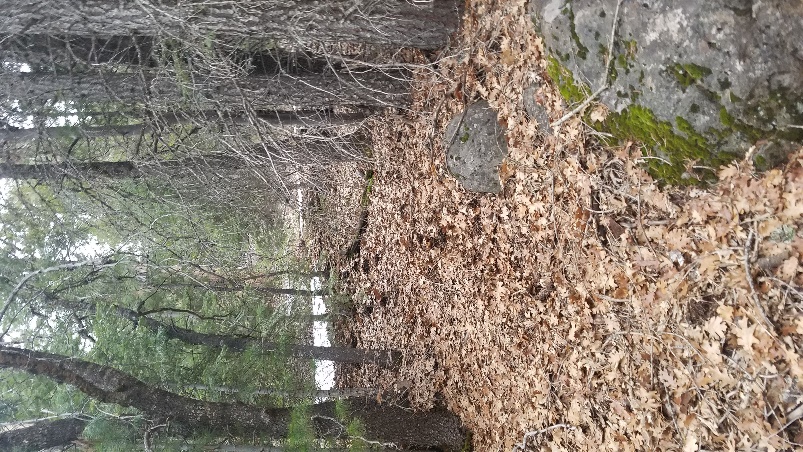

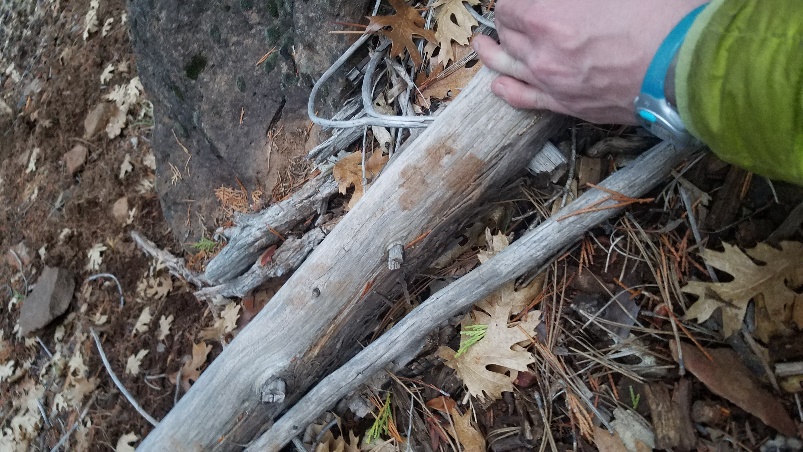

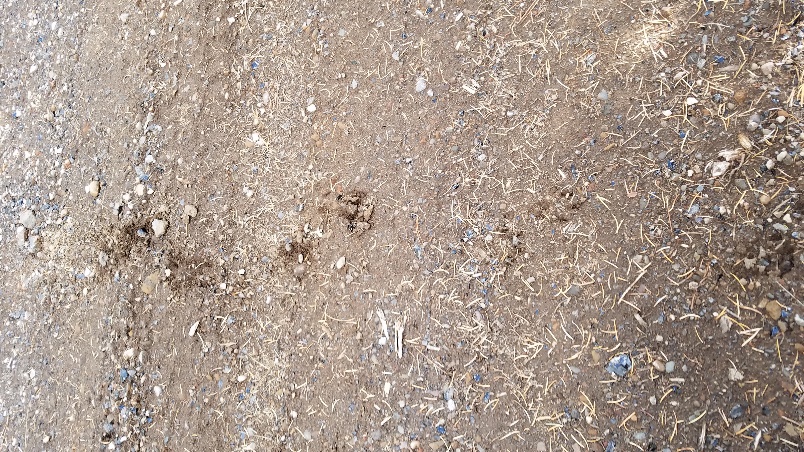


A

B

C

Fig. S1. Trail of a chase through the forest floor (A). The substrate was not conducive to identifying the predator involved in the chase, but carefully following the trail lead to the discovery of coyote tracks (B). A separate predation event with clear tracks indicating a coyote running. The tracks of a running adult female deer are also visible (C).


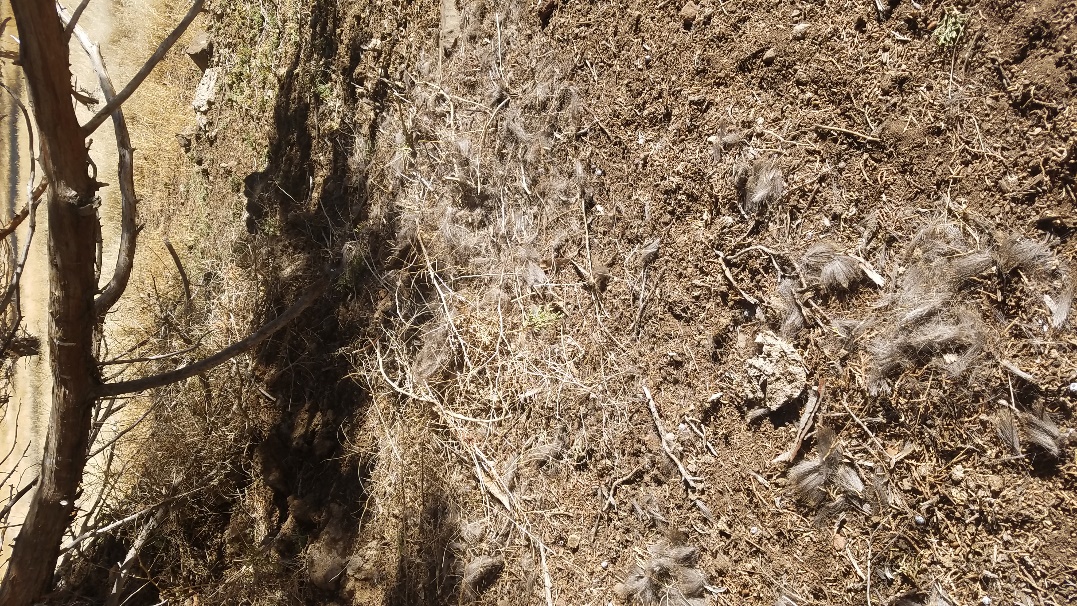

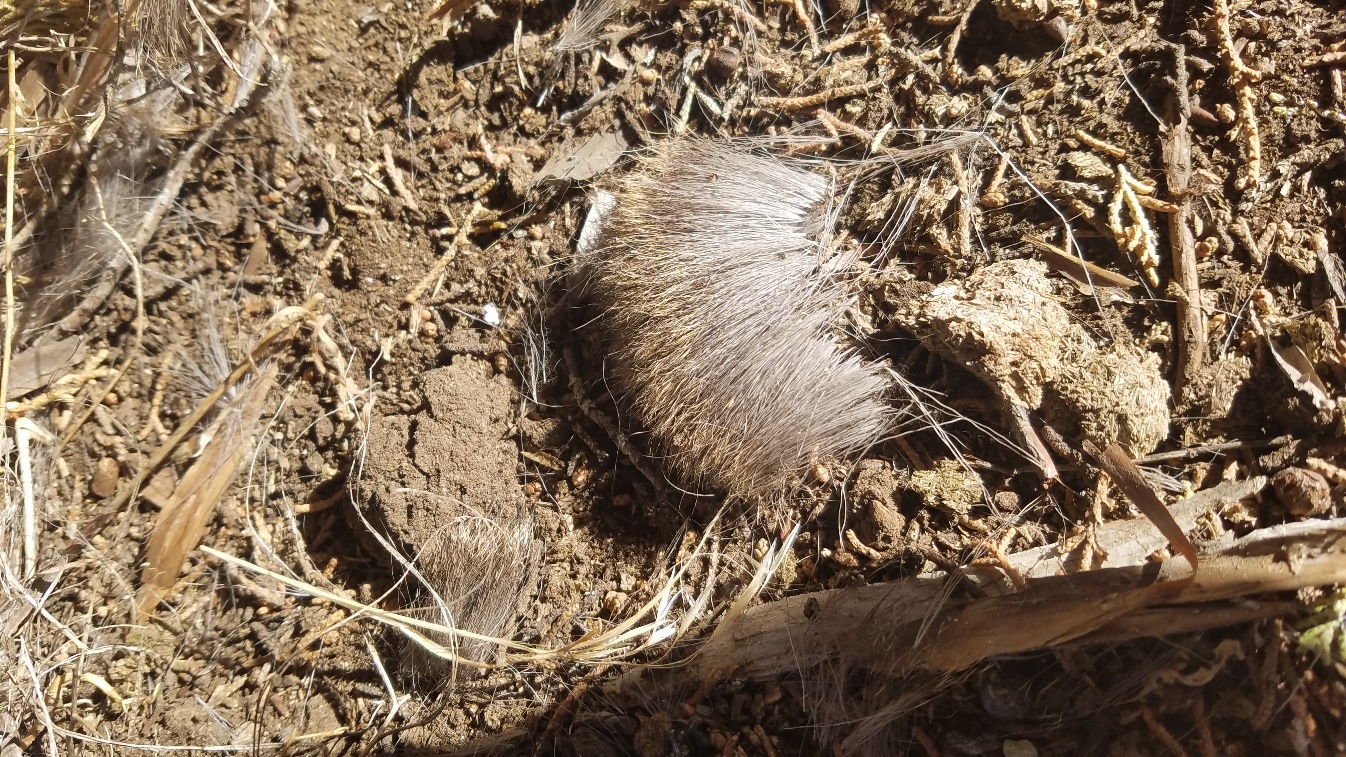

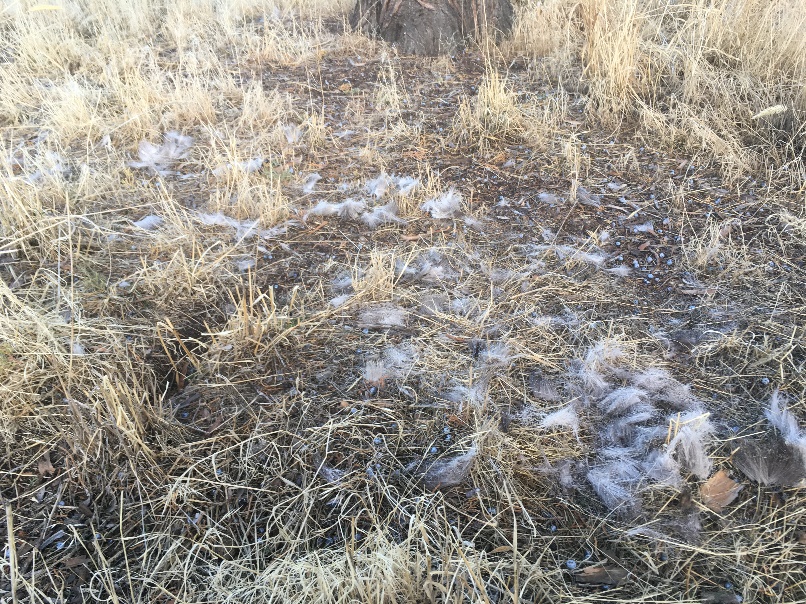

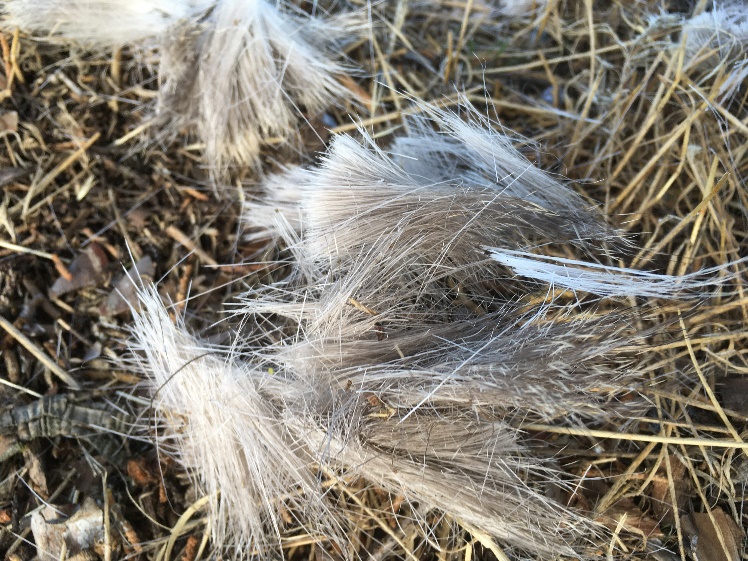


A

B

C

D

Fig. S2. Hair mat under a juniper tree (*Juniperus* spp.) (A) and sheared hair (B) located at a fawn mortality site, both indicative of carcass consumption by a Felid. Comparable evidence is presented for another fawn mortality site (C, D).


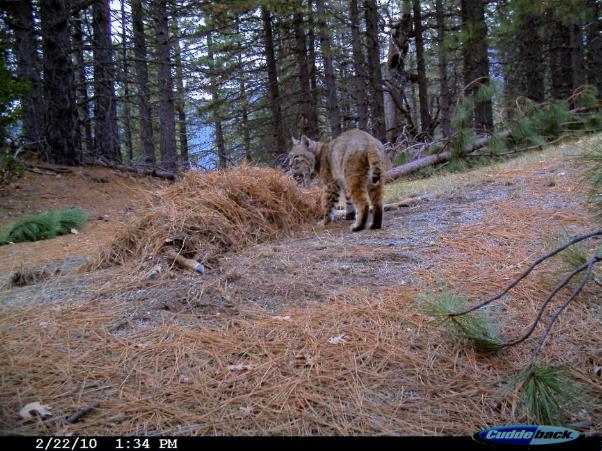


Fig. S3. Cache sites can be neatly piled if adequate substrate materials are available.


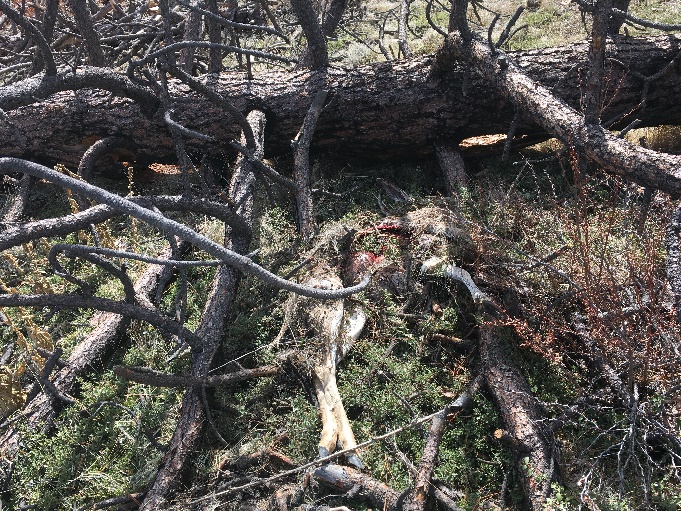

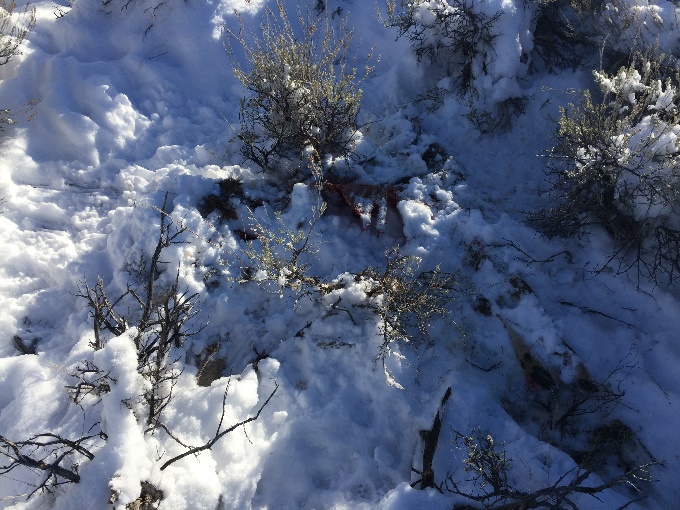


A

B

Fig. S4. Solitary felids may cache their prey (A) but also carcasses they come across, so caching alone should not be considered indicative of a predation event. Caching might occur more frequently in warm months of the year to prevent meat spoilage from heat and insects, as well to minimize detection by scavengers, but can occur in winter also (B).


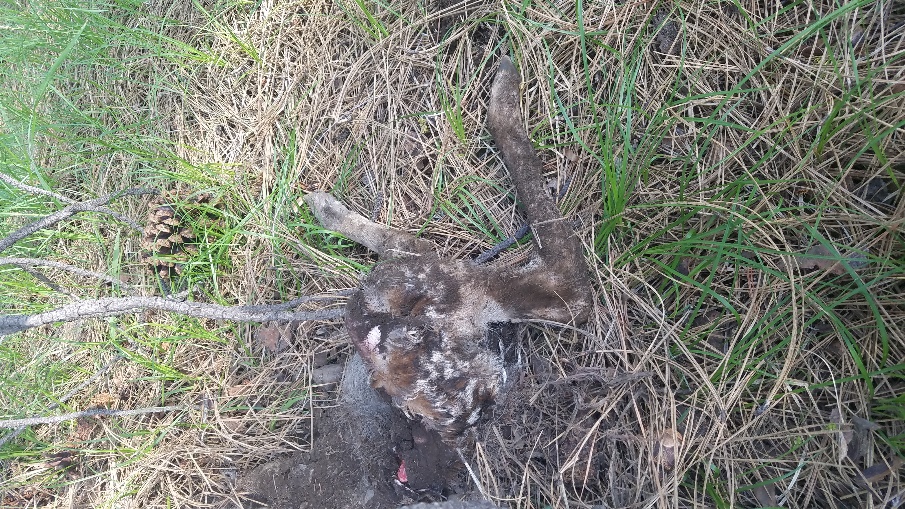

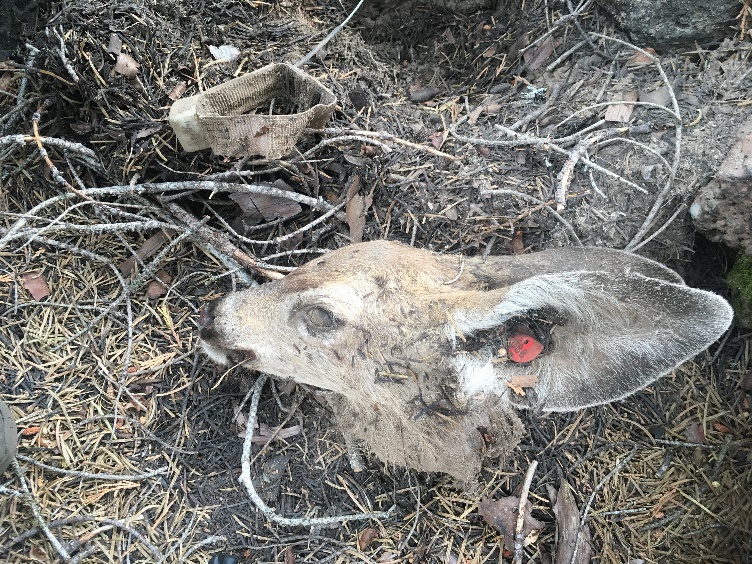

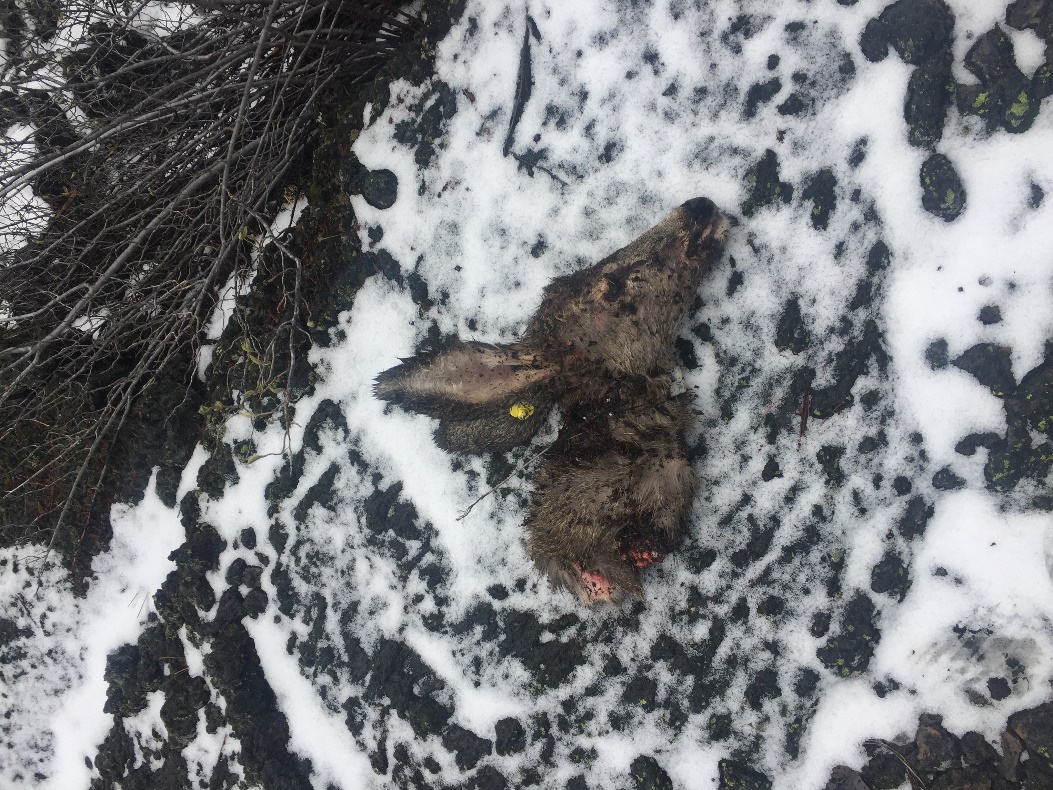


A


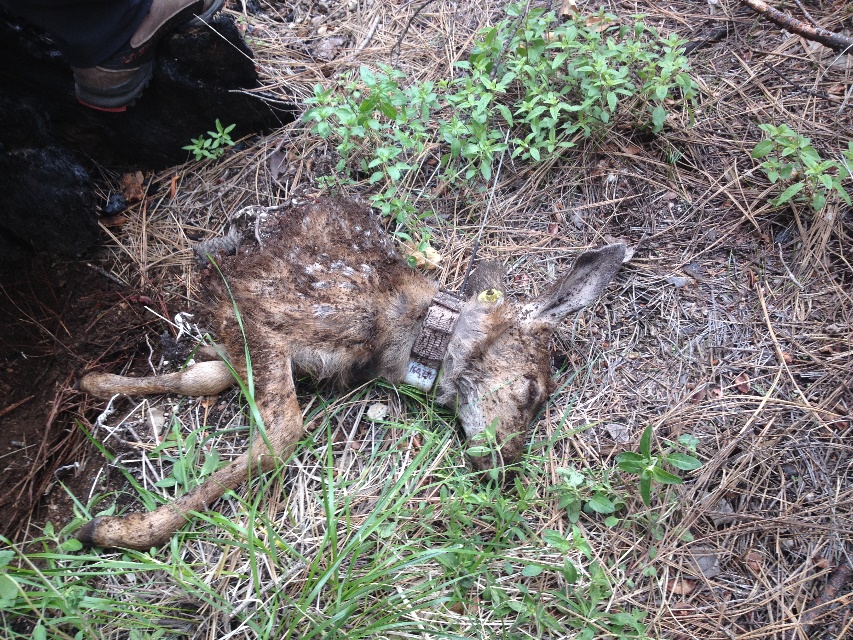


B

C

D

Fig. S5. Coyotes often de-articulate fawns and bury the body parts at distinct locations. Here the parts have been unearthed by field crews to inspect them for evidence of predation (A-D), including from marks on the hide and skinning to identify hematoma.


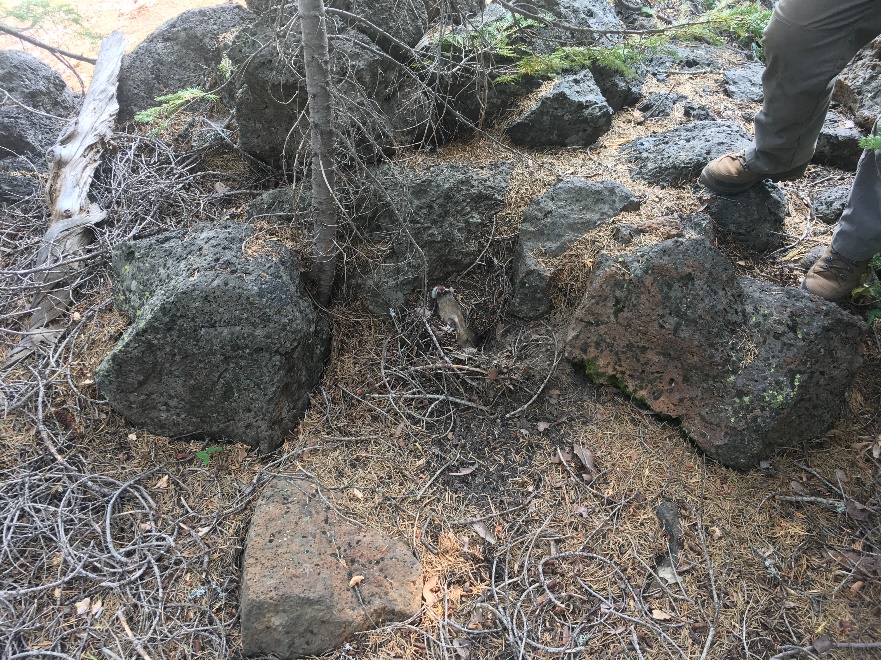

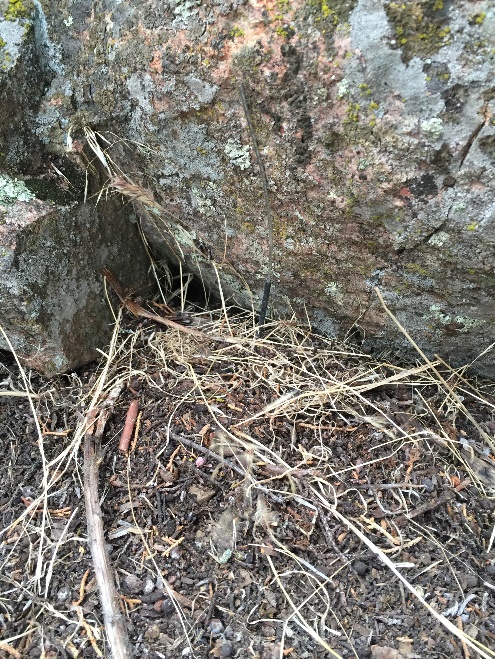

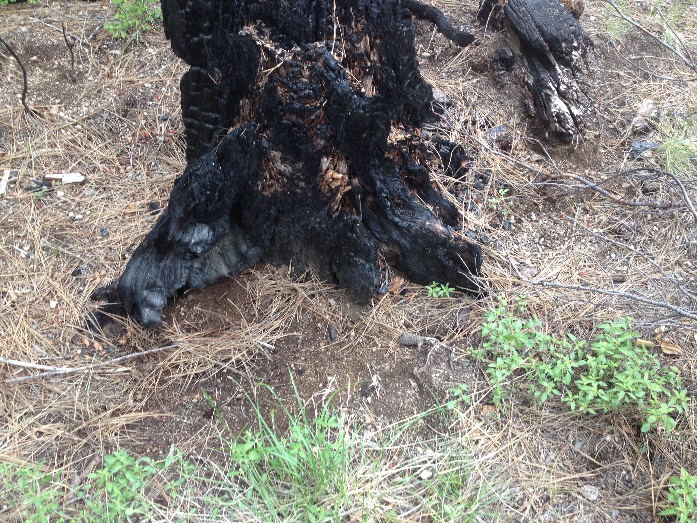

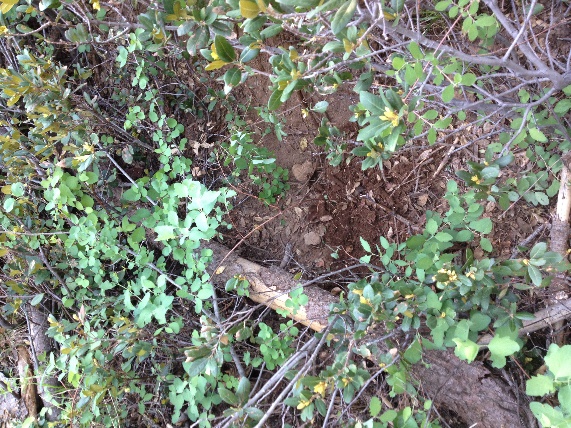

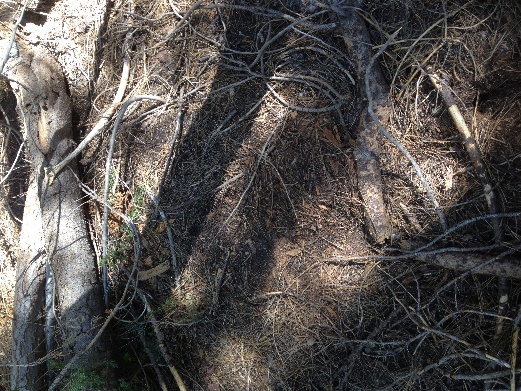


A

B

C

D

E

Fig. S6. Burial sites of fawns by coyotes sometimes leave a small part of the fawn’s body exposed (A), but more often are discrete and difficult to locate even with the aid of telemetry. Clues may include a collar antenna sticking out of the soil surface (B), or moist soil that could indicate a fresh burial (C-E).


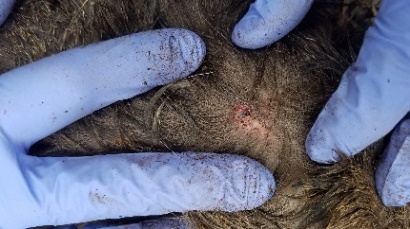

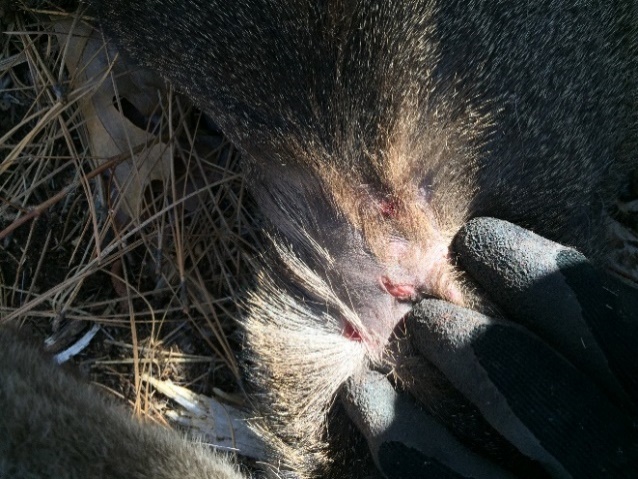

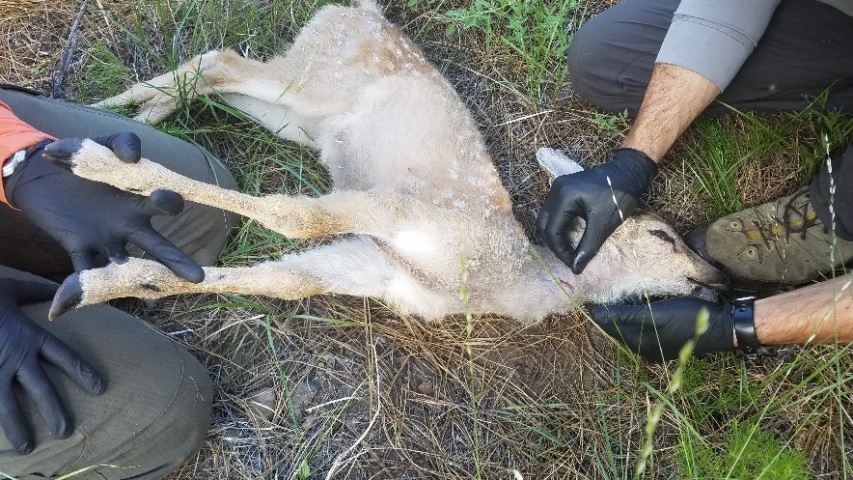

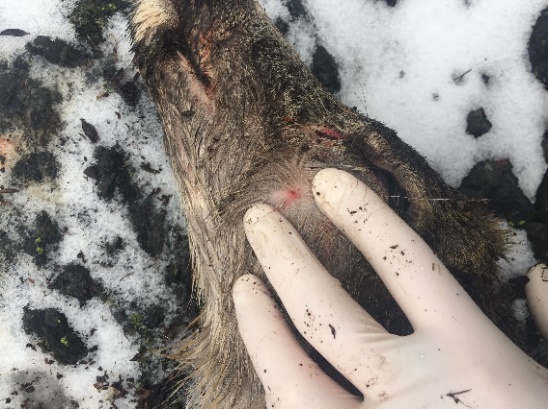

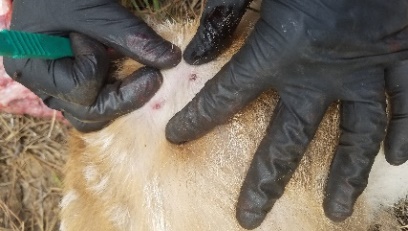

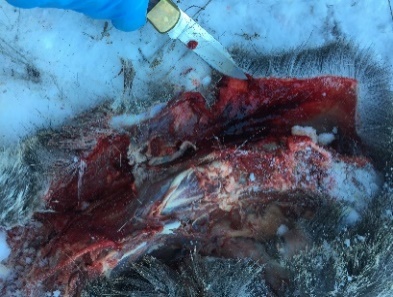

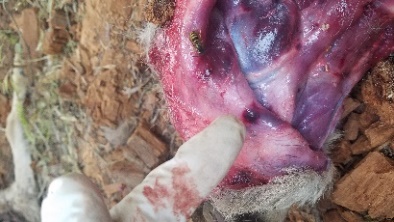

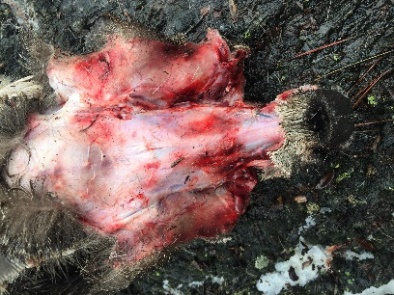

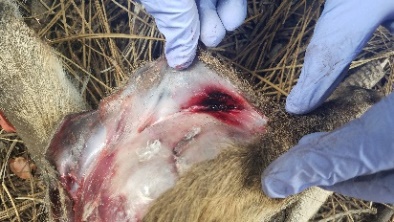

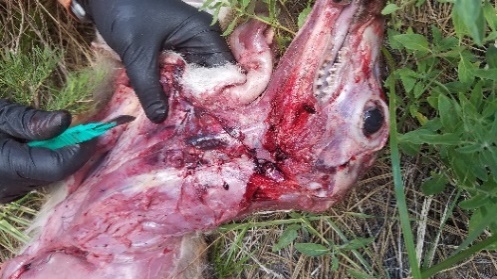


A

B

C

D

E

F

G

H

I

J

Fig. S7. Sometimes marks from predation are visible on the hide, such as canine bites or claw marks (A-E). However, skinning the carcass will typically reveal more information and hidden marks, such as punctures and impressions from claws (F-J).


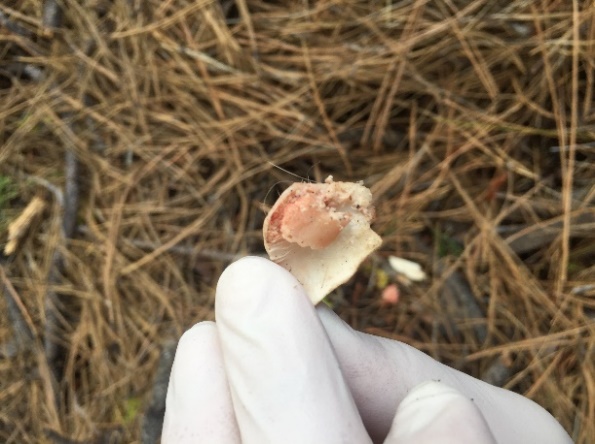

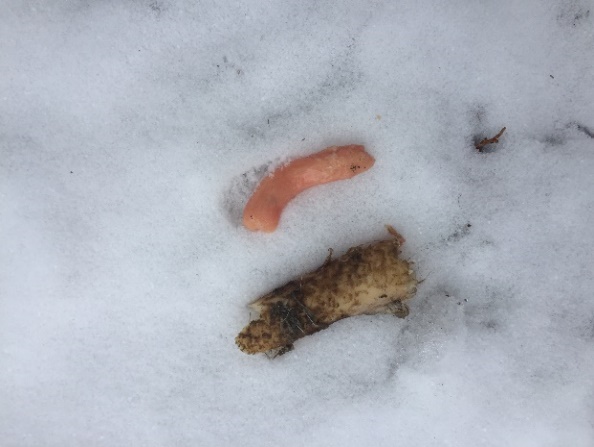

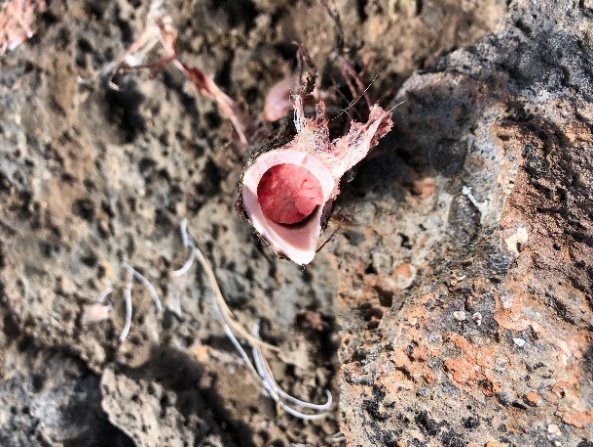

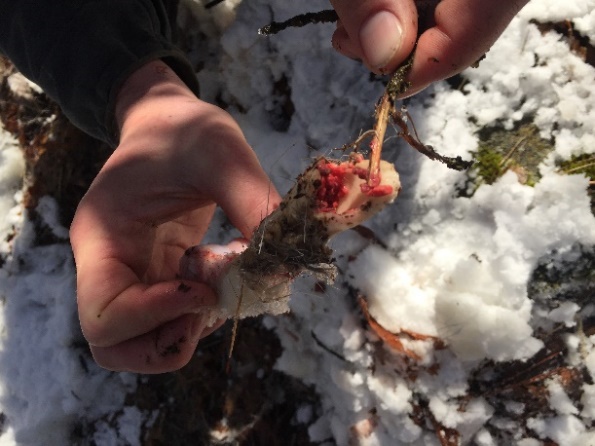

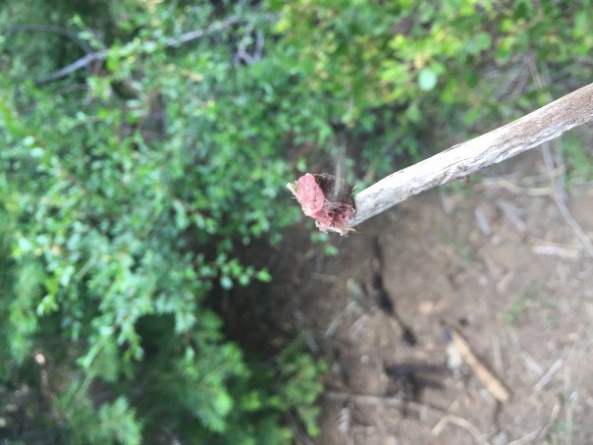

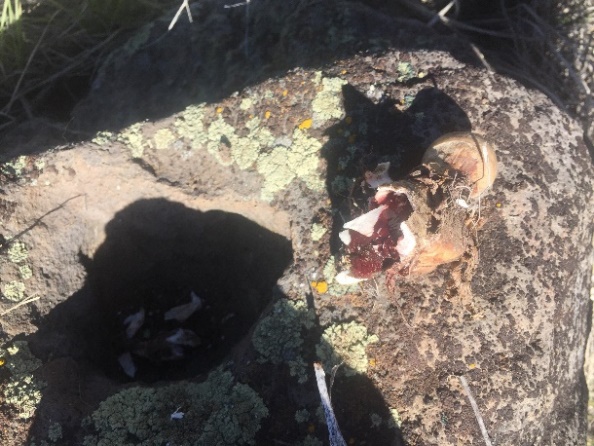


A

B

C

D

E

F

Fig. S8. The color and consistency of the marrow in long bones (e.g., femur or humerus) can be indicative of body condition of adult ungulates. Color varies on a gradient from white to red (A-F), with red indicating poorest fat reserves and thereby poorest condition. Consistency varies from solid to gelatinous, with the latter indicating poor fat reserves.


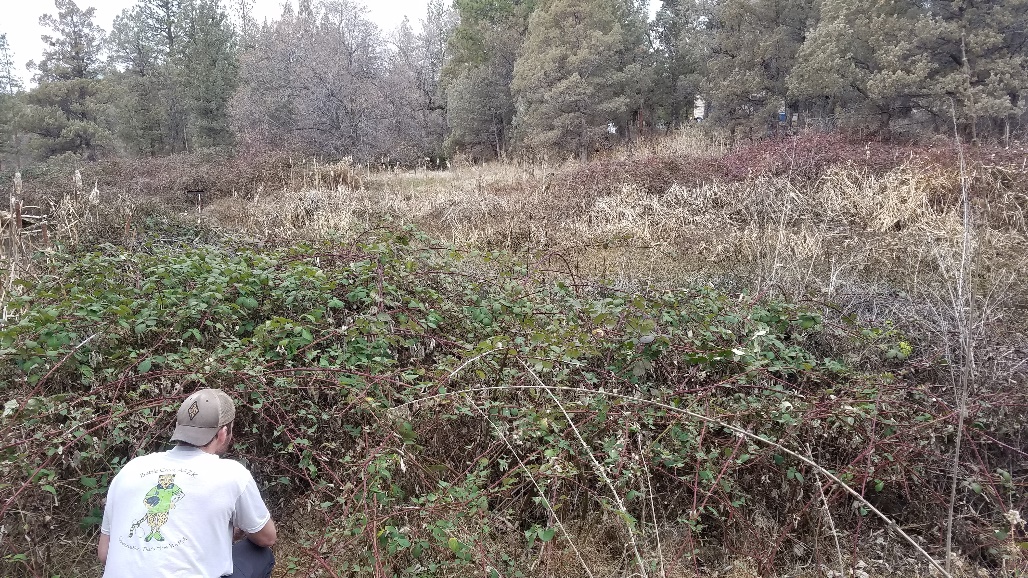

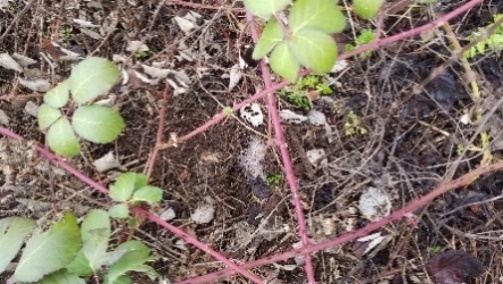

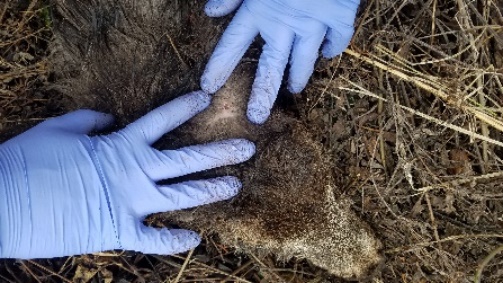

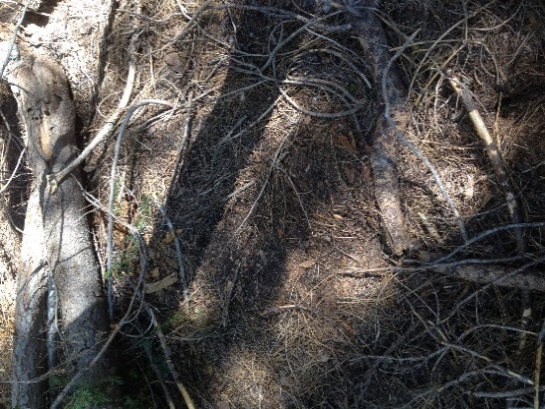

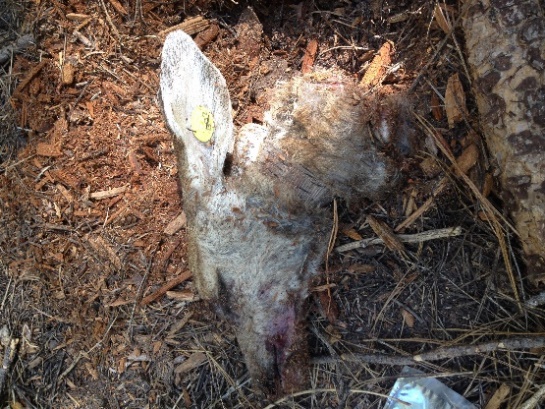

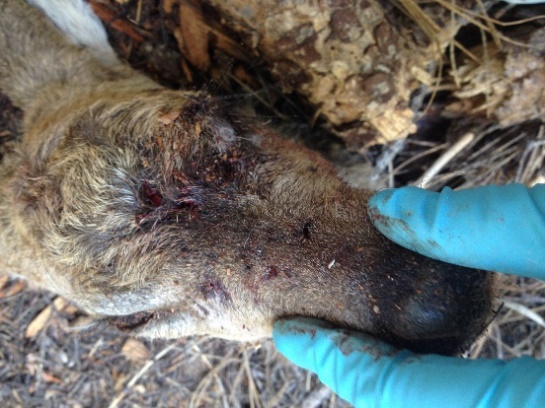

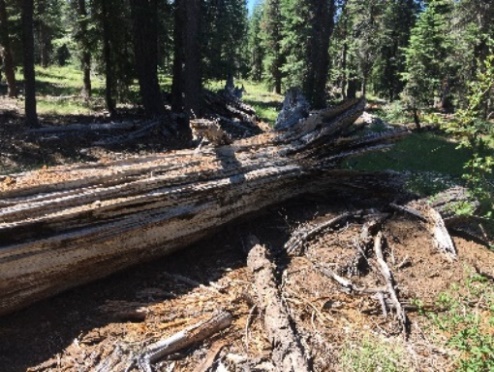

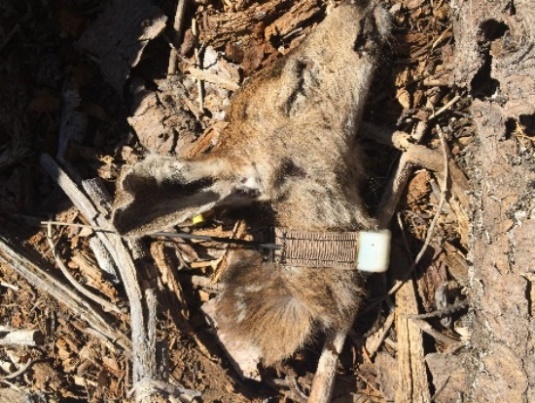

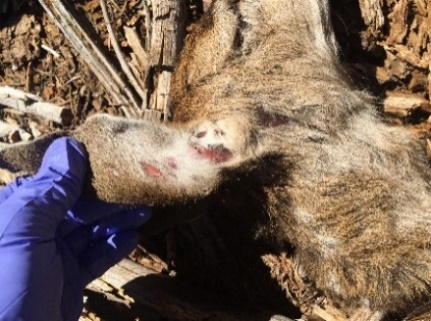


A

B

C

D

E

F

G

H

I

Fig. S9. Examples of coyote predation sites (general area and carcass). Photographs are grouped by predation event, with 3 events illustrated in total (A-C, D-F, G-I).


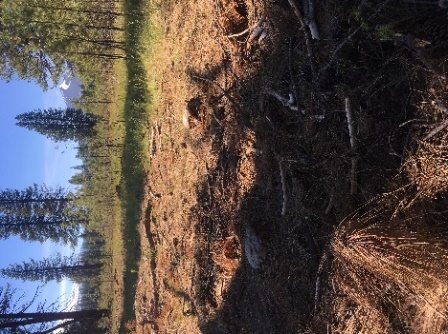

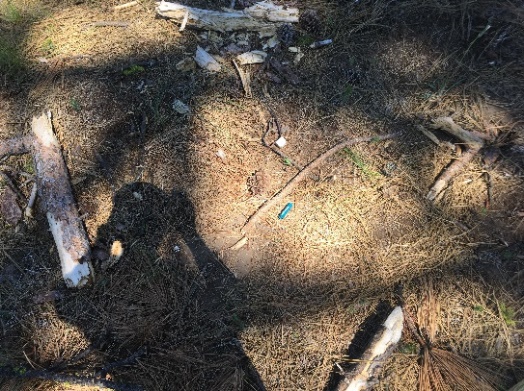

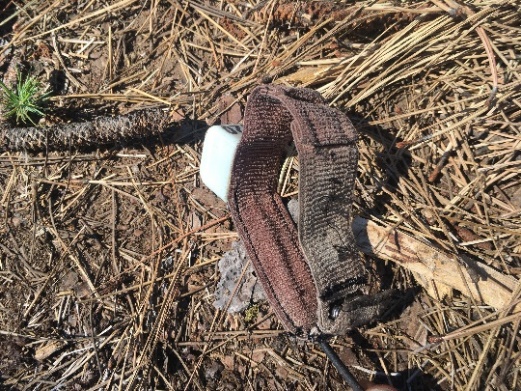

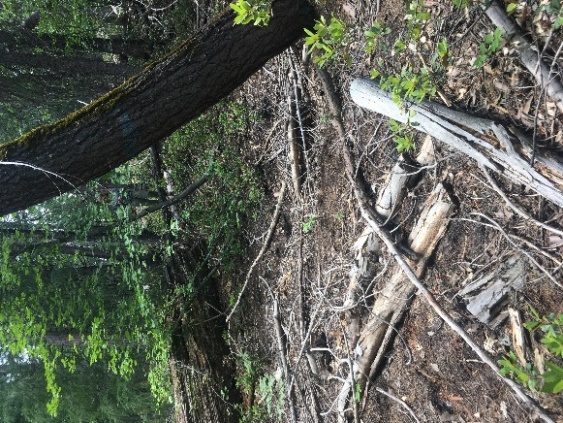

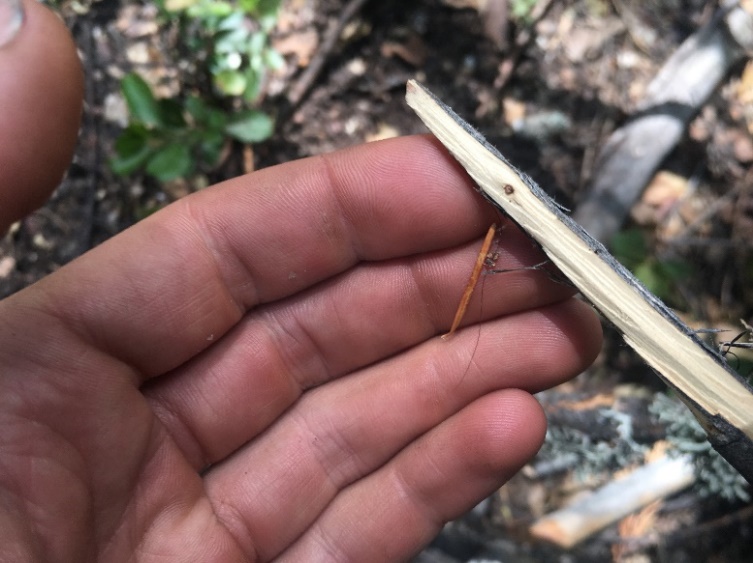

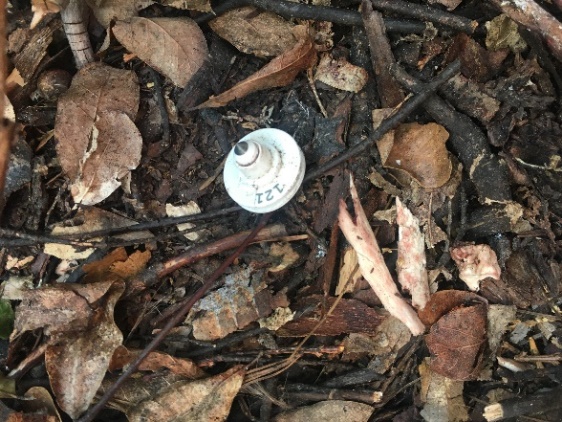

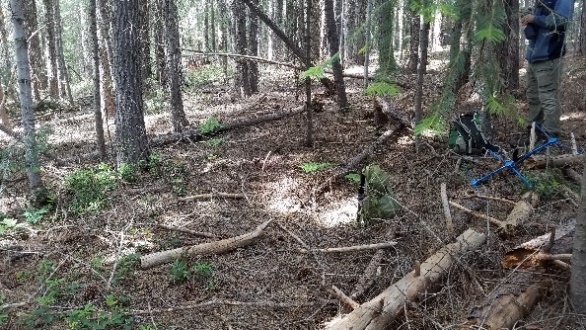

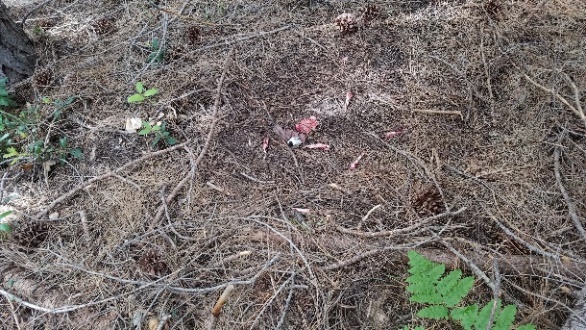

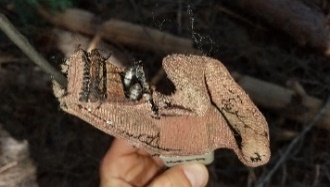


A

B

C

D

E

F

G

H

I

Fig. S10. Examples of black bear predation sites (general area and remnants). Photographs are grouped by predation event, with 3 events illustrated in total (A-C, D-F, G-I).


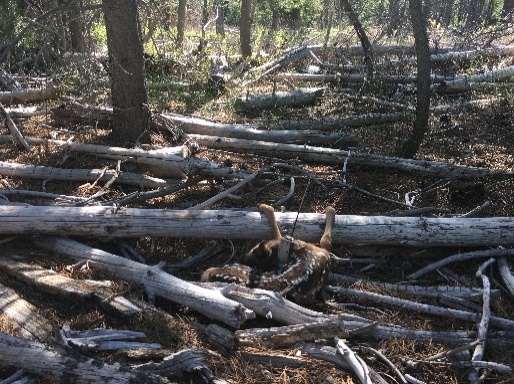

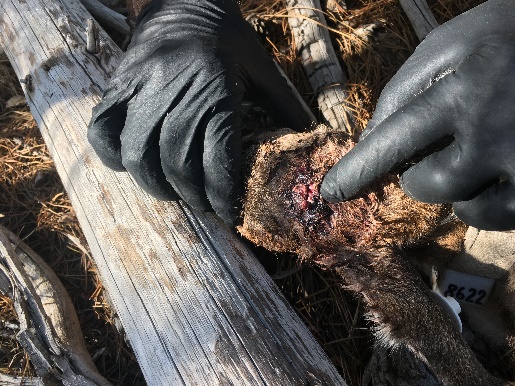

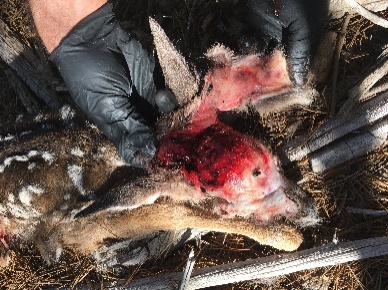

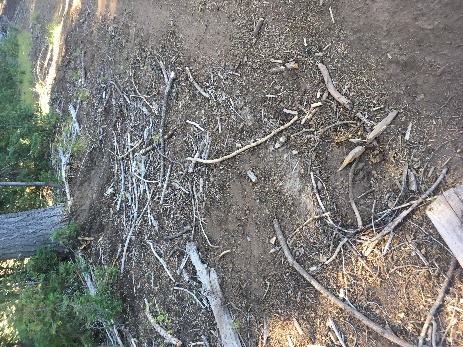

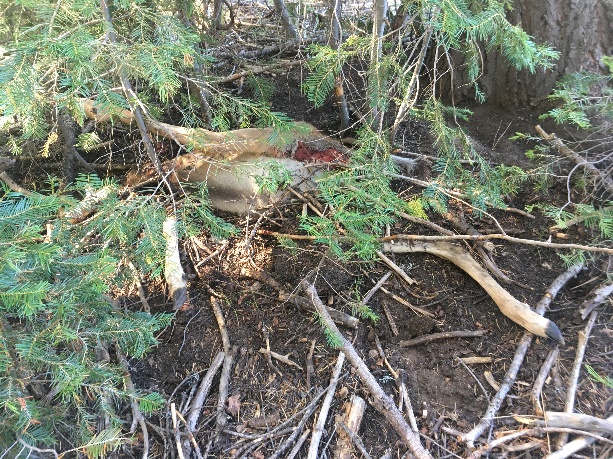

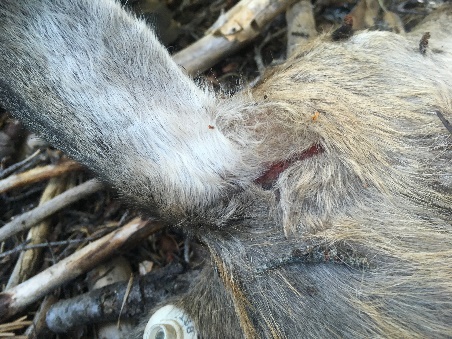

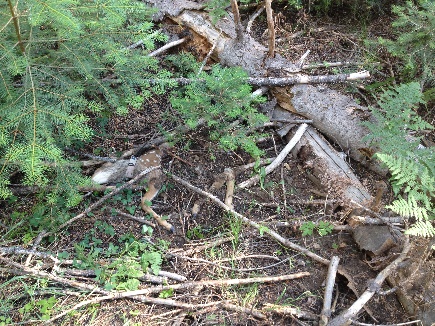

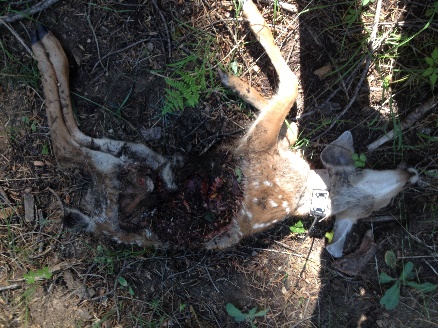

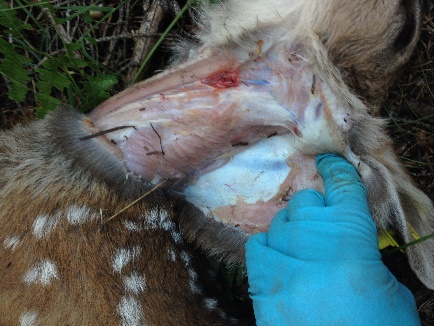


A

B

C

D

E

F

G

H

I

Fig. S11. Examples of bobcat predation sites (general area and carcass). Photographs are grouped by predation event, with 3 events illustrated in total (A-C, D-F, G-I).


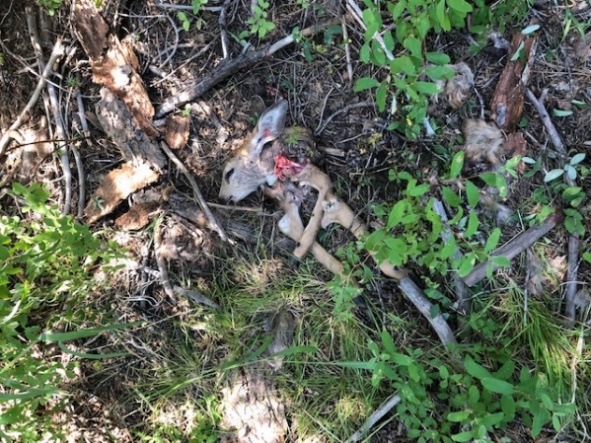

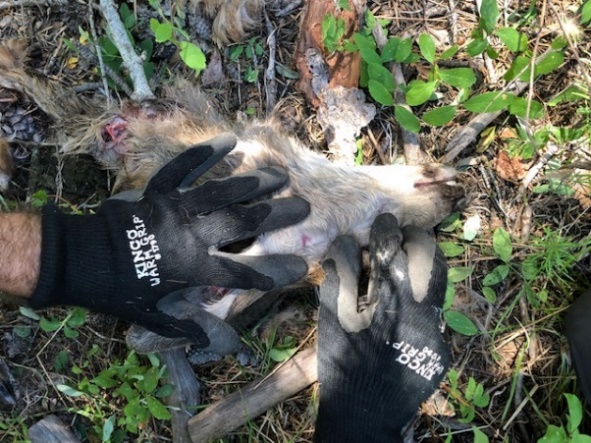

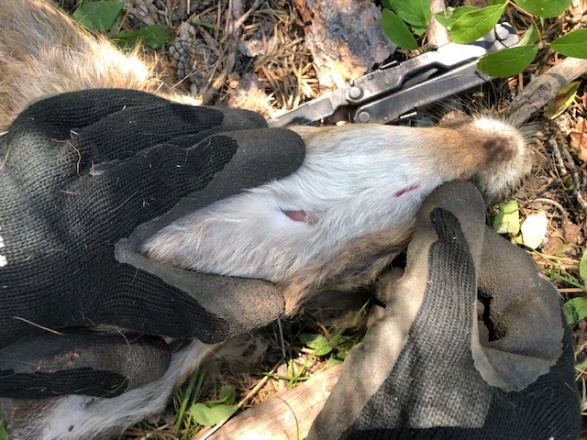

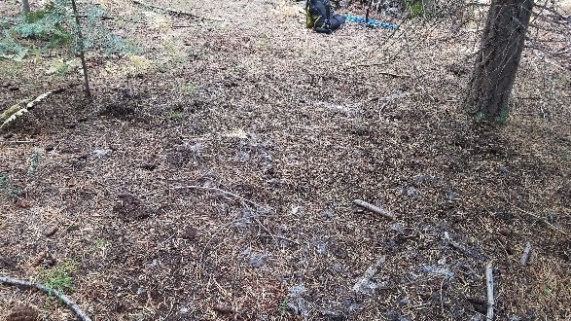

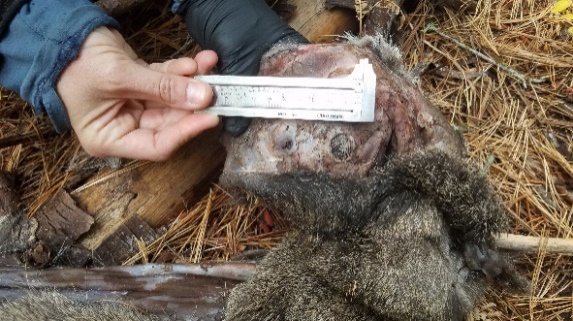

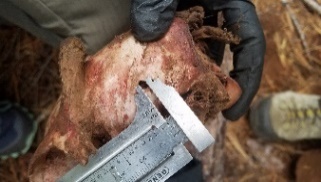

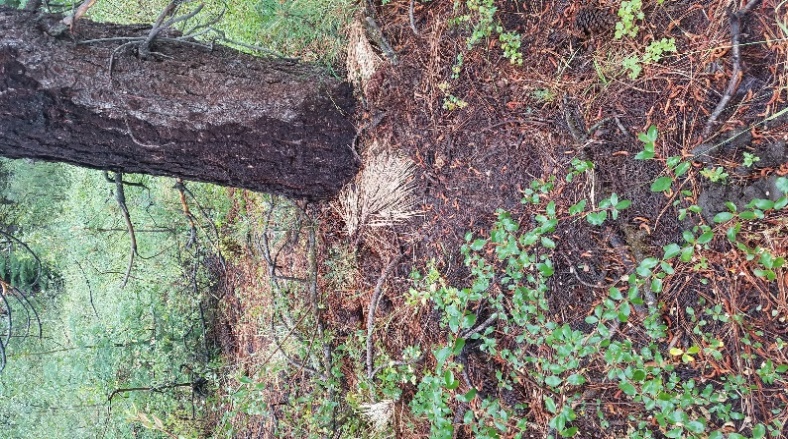

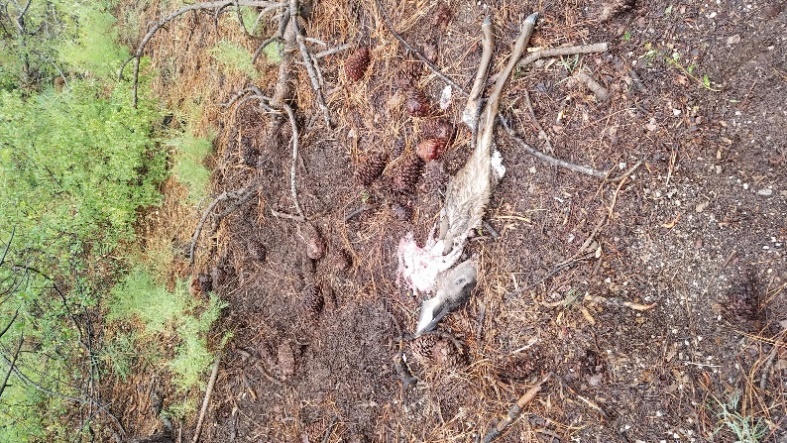

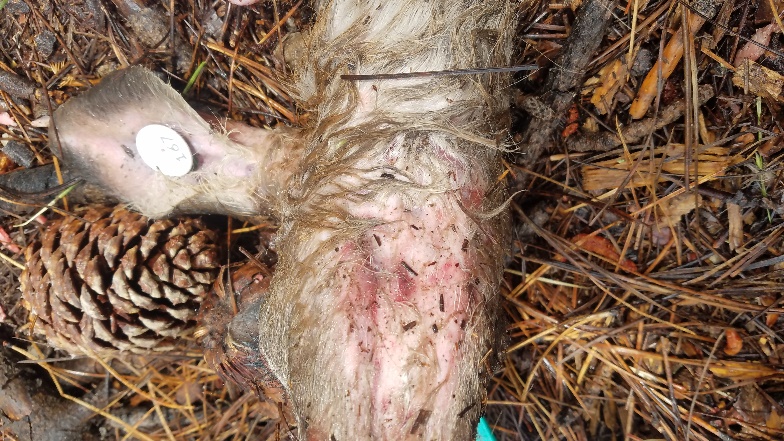


A

B

C

D

E

F

G

H

I

Fig. S12. Examples of puma predation sites on fawns (general area and carcass). Photographs are grouped by predation event, with 3 events illustrated in total (A-C, D-F, G-I).


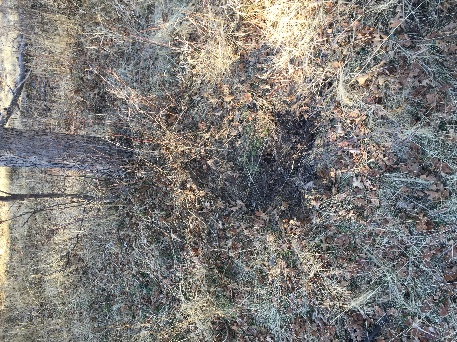

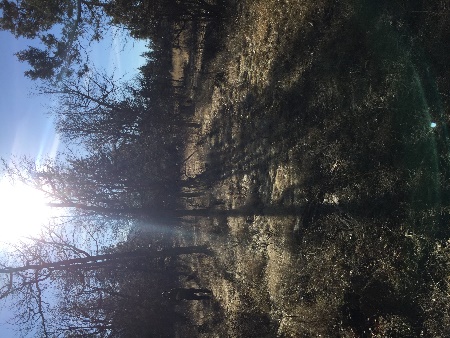

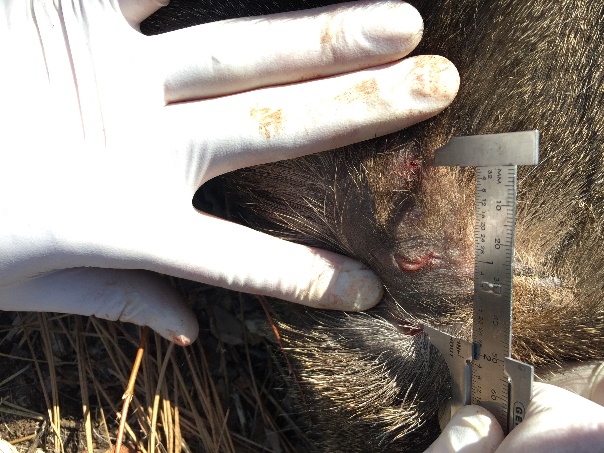

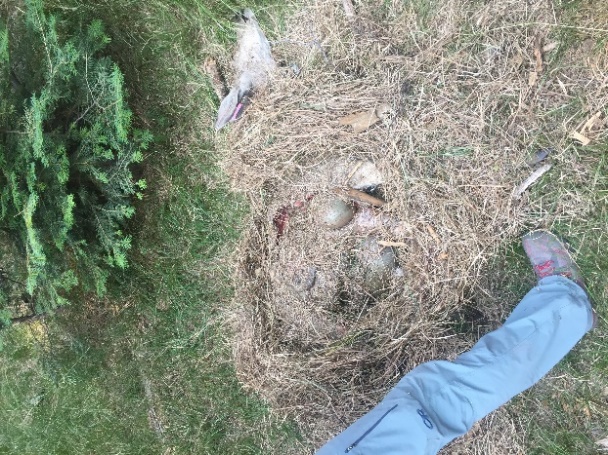

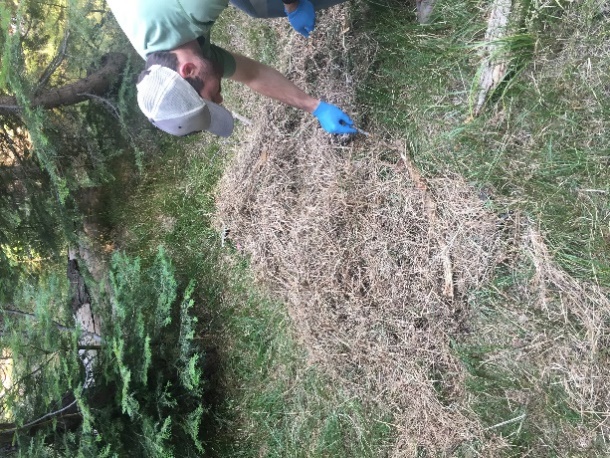

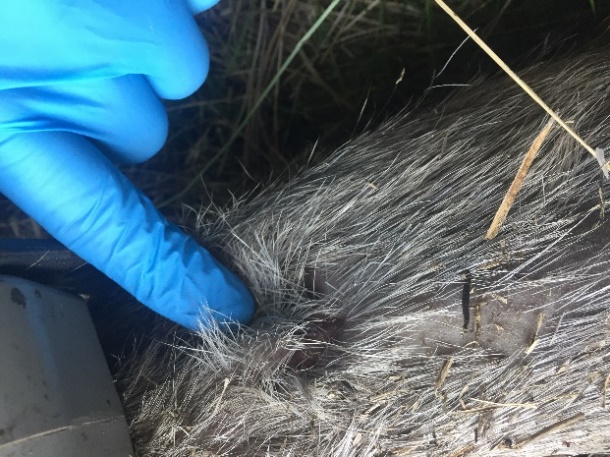

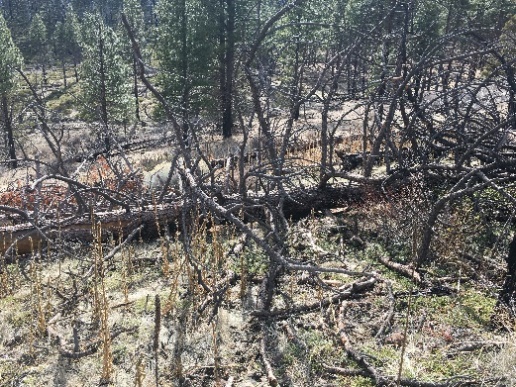

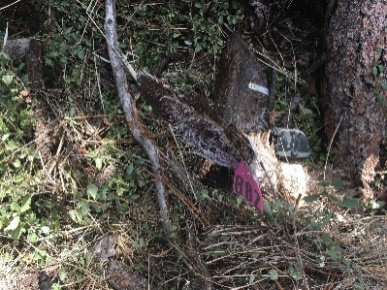

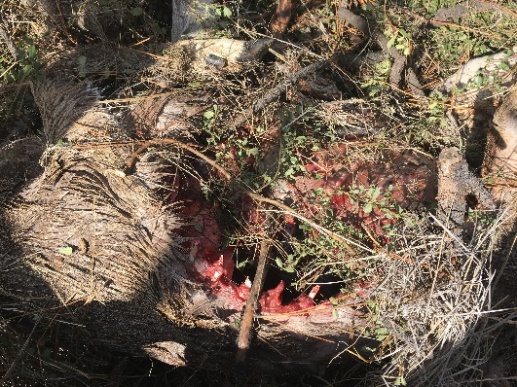


A

C

D

E

F

G

H

I

B

Fig. S13. Examples of puma predation sites on adult deer (general area and carcass). Photographs are grouped by predation event, with 3 events illustrated in total (A-C, D-F, G-I).
