# Supplementary material for: Standardizing protocols for determining the cause of mortality in wildlife studies
Source: Ecol Evol. 2022 Jun 23;12(6):e9034. doi: 10.1002/ece3.9034 (PMC9219102; doi:10.1002/ece3.9034)
Supplement: Supplementary file 4 — Appendix S4 [file ECE3-12-e9034-s006.docx]

**Appendix S4**

Cristescu, B., L. M. Elbroch, T. D. Forrester, M. L. Allen, D. B. Spitz, C. C. Wilmers, and H. U. Wittmer. Standardizing protocols for determining the cause of mortality in wildlife studies. Ecology and Evolution.

Table S1. Number of mortality events for marked mule deer fawns in northern California (2016-2020), along with mortality cause and confidence level in cause of death assignment. Data in brackets represent percentages relative to the total number of mortalities recorded.

|  | **Mortality cause** | | | |
| --- | --- | --- | --- | --- |
|  | **Low** | **Medium** | **High** | **Pooled** |
| Predation | 2 (2) | 34 (37) | 34 (37) | 70 (76) |
| Malnutrition/Disease | 0 (0) | 0 (0) | 0 (0) | 0 (0) |
| Natural hazard | 0 (0) | 0 (0) | 2 (2) | 2 (2) |
| Roadkill | 0 (0) | 0 (0) | 3 (3) | 3 (3) |
| Unknown | 16 (18) | 0 (0) | 0 (0) | 16 (18) |

Table S2. Number of mortality events for marked mule deer >1 year old in northern California (2016-2020), along with mortality cause and confidence level in cause of death assignment. Data in brackets represent percentages relative to the total number of mortalities recorded. Earlier data (2015) were collected opportunistically and are therefore not included.

|  | **Mortality cause** | | | |
| --- | --- | --- | --- | --- |
|  | **Low** | **Medium** | **High** | **Pooled** |
| Predation | 0 (0) | 9 (39) | 7 (30) | 16 (69) |
| Malnutrition/Disease | 0 (0) | 1 (4) | 1 (4) | 2 (8) |
| Natural hazard | 0 (0) | 0 (0) | 0 (0) | 0 (0) |
| Roadkill | 0 (0) | 0 (0) | 0 (0) | 0 (0) |
| Unknown | 5 (22) | 0 (0) | 0 (0) | 5 (22) |
