# Supplementary material for: Standardizing protocols for determining the cause of mortality in wildlife studies
Source: Ecol Evol. 2022 Jun 23;12(6):e9034. doi: 10.1002/ece3.9034 (PMC9219102; doi:10.1002/ece3.9034)
Supplement: Supplementary file 5 — Appendix S5 [file ECE3-12-e9034-s004.docx]

**Appendix S5**

Cristescu, B., L. M. Elbroch, T. D. Forrester, M. L. Allen, D. B. Spitz, C. C. Wilmers, and H. U. Wittmer. Standardizing protocols for determining the cause of mortality in wildlife studies. Ecology and Evolution.

| Investigator(s) | Date of Investigation | | GPS Location | Snow Cover *(circle)* | Animal ID |
| --- | --- | --- | --- | --- | --- |
|  |  | | N  W | 0-25 / 25-50 /  50-75 / 75-100 |  |
|  | Time |  |  |  |  |

| 1. ***Discovery: Photos*** | | | | |
| --- | --- | --- | --- | --- |
| Take Photos Before  Moving Anything! | Camera ID | Datasheet Header *(tick)* | General Site *(tick)* | Carcass *(tick)* |
|  |  |  |  |  |

| 1. ***Site analysis*** | | | |
| --- | --- | --- | --- |
| **Drag Marks** | | | |
| Present? | Blood Spatter? | Disturbed Soil? | Broken/Flattened Vegetation? |
|  |  |  |  |
| **Tracks** | | | |
| Species | Behavior from Tracks | | Tracking Substrate |
|  |  | |  |
| **Scat** | | | |
| Species | Number of scats | | Freshness |
|  |  | |  |
| **Hair** | | | |
| Species | Location of Hair | | Sheared Hair? |
|  |  | |  |
| **Other Sign** | | | |
| Bed Sites | Rubs, Scrapes and Scratches | | Other |
|  |  | |  |

| 1. ***Carcass analysis*** | | | | | |
| --- | --- | --- | --- | --- | --- |
| Estimated Days Dead | | Carcass Length × Width | | | Disturbance Length × Width |
|  | |  | | |  |
| **Cached and Buried Remains** | | | | | |
| Cached? | | Buried? | | | Gut Pile? |
|  | |  | | |  |
| **State and Placement** | | | | | |
| Blood | Leg Position *(circle)* | Entrance Point(s) | | Parts Consumed | % Consumed *(circle)* |
|  | Under / Side |  | |  | 0-25 / 25-50 / 50-75 / 75-100 |
| **Necropsy and Carcass Details** | | | | | |
| Bite/Tooth Puncture | | Tooth Puncture Diameter (mm) | | | Inter-canine Distance (mm) |
|  | |  | | |  |
| Marks on Hide | | Blood cloth/Bruises upon Skinning | | | Location of Blood Cloth/Bruises |
|  | |  | | |  |
| Bone Condition | | Ribs Partially Broken/Chewed | Marrow Consistency *(circle)* | | Marrow Color *(circle)* |
|  | |  | Solid / Liver / Gelatinous | | White / White-Pink / Pink-Red / Red / Spotted / Yellow |
| **Samples** *(circle)* | | | | | |
| Brain / DNA Swab / Ectoparasite / Femur / Hair / Humerus / Mandible / Scat / Teeth / Tissue / Other  Sample comments: | | | | | |

| 1. ***Habitat analysis*** | | | | | | | | | |
| --- | --- | --- | --- | --- | --- | --- | --- | --- | --- |
| **Kill Site** | | | | | | | | | |
| Slope | Aspect | Dominant Tree Species | | | | Dominant Shrub Species | | | |
|  |  |  | | | |  | | | |
| Horizontal Cover | | | | | | Vertical Cover | | | |
| *Direction* | | N | E | S | W | N | E | S | W |
| Shrub / Tree | |  |  |  |  |  |  |  |  |
| High Ground | |  |  |  |  |  | | | |
| Low Ground | |  |  |  |  |  |  |  |  |
| **Mortality Site** | | | | | | | | | |
| Slope | Aspect | Dominant Tree Species | | | | Dominant Shrub Species | | | |
|  |  |  | | | |  | | | |
| Horizontal Cover | | | | | | Vertical Cover | | | |
| *Direction* | | N | E | S | W | N | E | S | W |
| Shrub / Tree | |  |  |  |  |  |  |  |  |
| High Ground | |  |  |  |  |  | | | |
| Low Ground | |  |  |  |  |  |  |  |  |

| **Map of Site and Comments:** |
| --- |
