# Supplementary figures and images for: Standardizing protocols for determining the cause of mortality in wildlife studies
Source: Ecol Evol. 2022 Jun 23;12(6):e9034. doi: 10.1002/ece3.9034 (PMC9219102; doi:10.1002/ece3.9034)

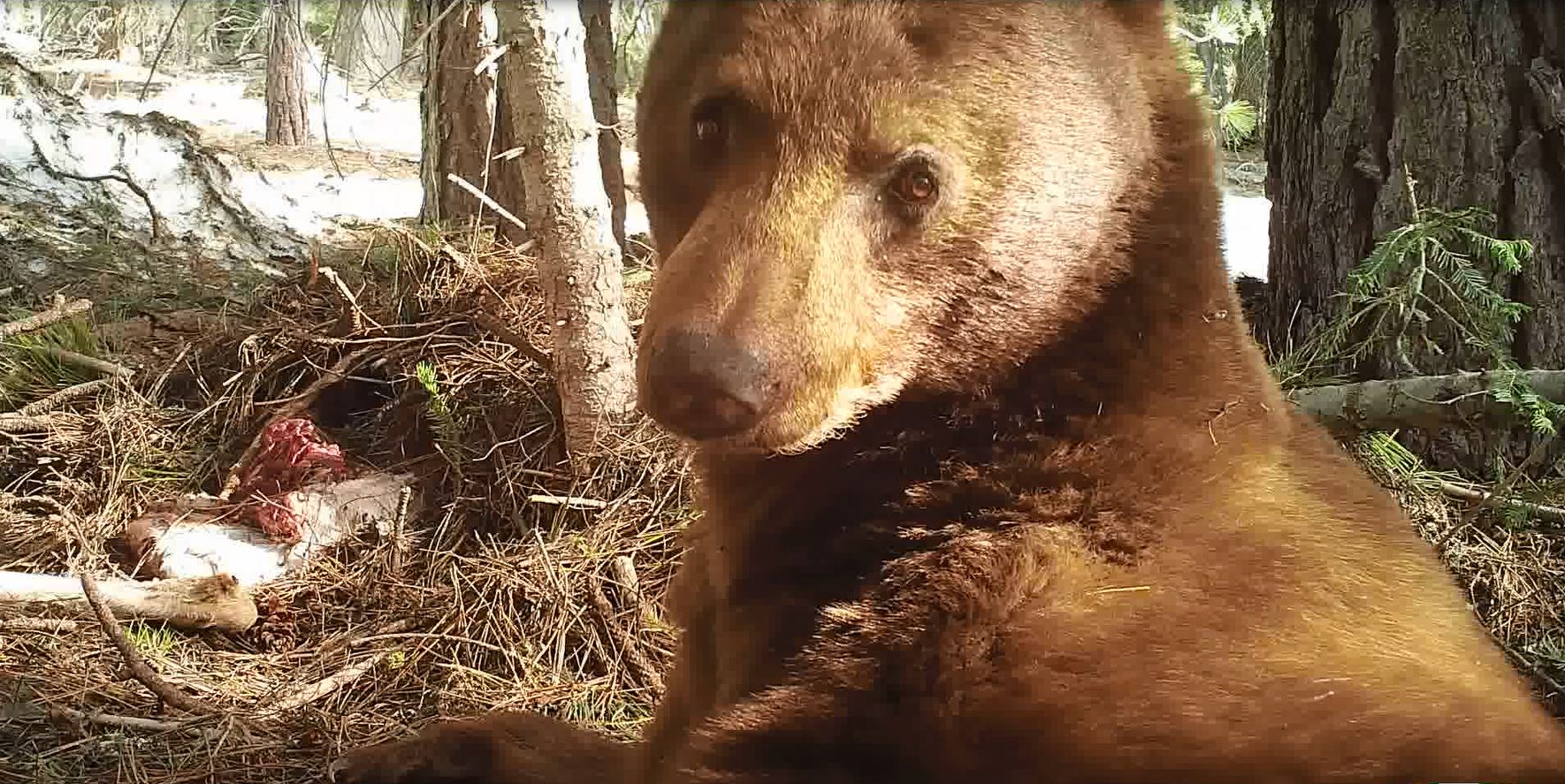

Supplement: Supplementary file 6 — Video S1 [file ECE3-12-e9034-s007.zip › PlaceholdeImage_VideoS1.JPG]

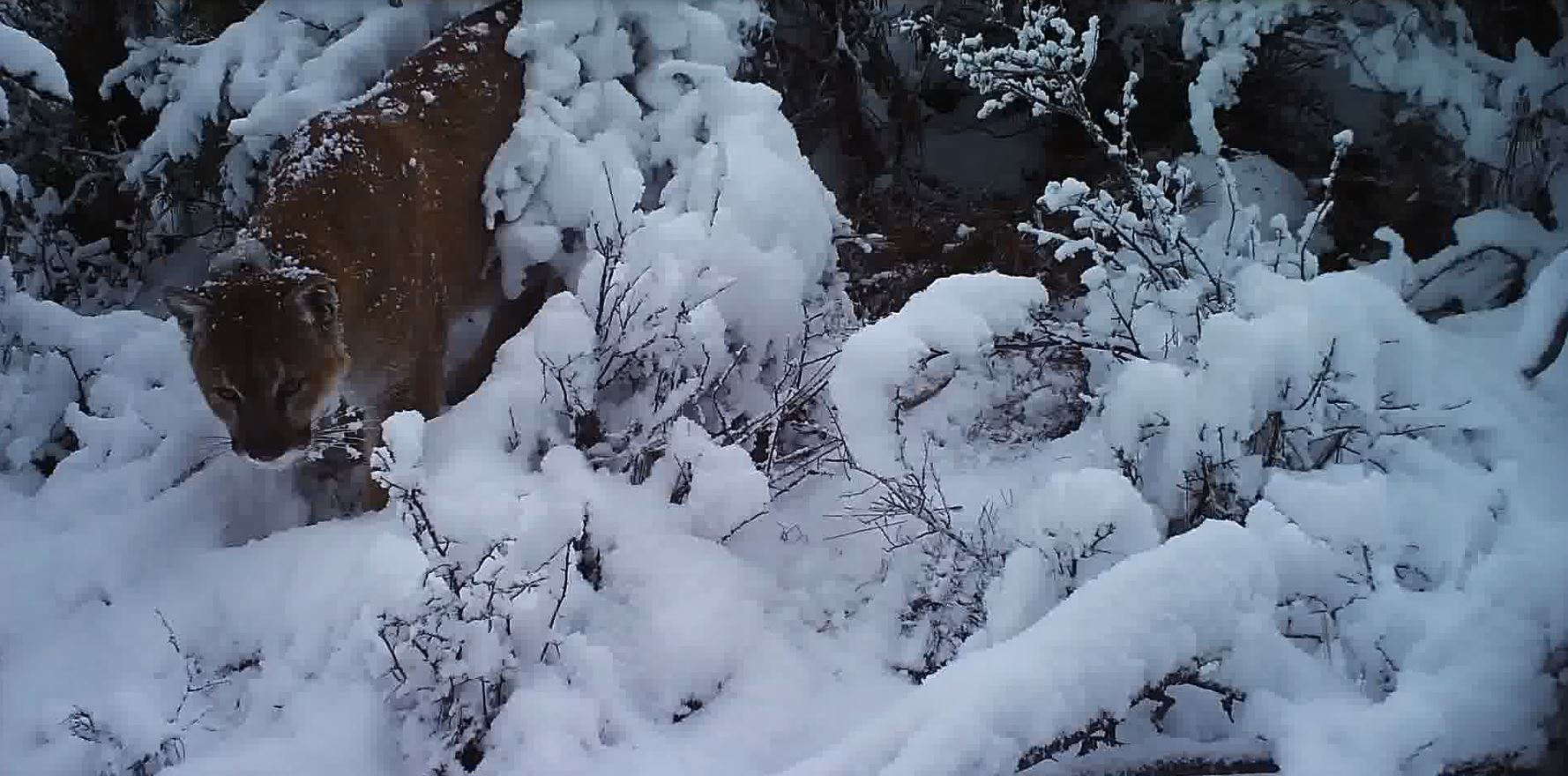

Supplement: Supplementary file 7 — Video S2 [file ECE3-12-e9034-s003.zip › PlaceholdeImage_VideoS2.JPG]
